# Proximal Cysteines that Enhance Lysine *N*-Acetylation of Cytosolic Proteins in Mice Are Less Conserved in Longer-Living Species

**DOI:** 10.1016/j.celrep.2018.07.007

**Published:** 2018-08-07

**Authors:** Andrew M. James, Anthony C. Smith, Cassandra L. Smith, Alan J. Robinson, Michael P. Murphy

**Affiliations:** 1 Medical Research Council Mitochondrial Biology Unit, University of Cambridge, Cambridge CB2 0XY, UK

## Abstract

Acetyl-coenzyme A (CoA) is an abundant metabolite that can also alter protein function through non-enzymatic *N*-acetylation of protein lysines. This *N-*acetylation is greatly enhanced *in vitro* if an adjacent cysteine undergoes initial *S-*acetylation, as this can lead to *S→N* transfer of the acetyl moiety. Here, using modeled mouse structures of 619 proteins *N*-acetylated in mouse liver, we show lysine *N-*acetylation is greater *in vivo* if a cysteine is within ∼10 Å. Extension to the genomes of 52 other mammalian and bird species shows pairs of proximal cysteine and *N-*acetylated lysines are less conserved, implying most *N-*acetylation is detrimental. Supporting this, there is less conservation of cytosolic pairs of proximal cysteine and *N-*acetylated lysines in species with longer lifespans. As acetyl-CoA levels are linked to nutrient supply, these findings suggest how dietary restriction could extend lifespan and how pathologies resulting from dietary excess may occur.

## Highlights

• Creation of a mouse structural library of *in vivo* lysine *N-*acetylation on 619 proteins
• Proximal cysteines enhance protein lysine *N-*acetylation *in vivo*
• Proximal *N-*acetylated lysine and cysteine residues are less conserved
• Conservation of these proximal residues is lower in species with longer lifespans

## Graphical Abstract

**Figure:**
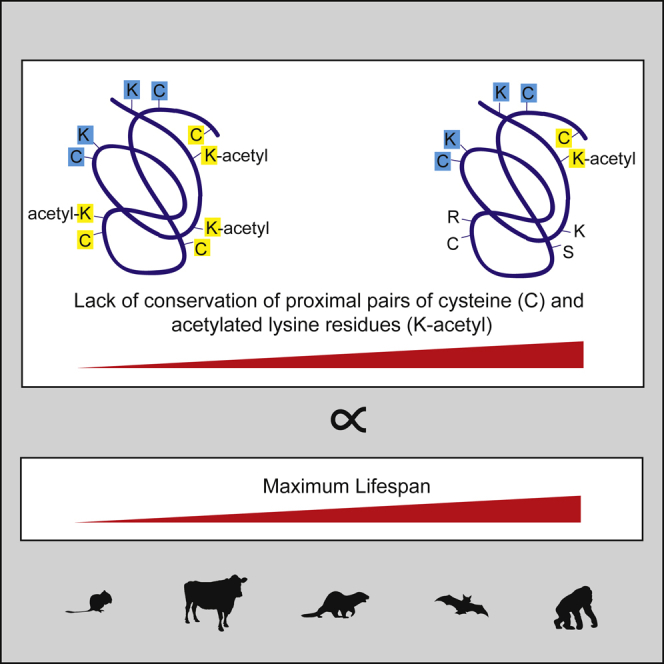

## Teaser

Acetyl-CoA non-enzymatically *N*-acetylates protein lysines. Using proteins *N*-acetylated in mouse liver, James et al. show *N-*acetylation is greater if a cysteine is within ∼10 Å. These pairs of proximal cysteine and *N-*acetylated lysines are less conserved in species with longer lifespans. This might explain how dietary restriction extends lifespan.

## Introduction

Acetyl-coenzyme A (CoA) and other acyl-CoAs are central metabolites in the oxidation of carbohydrate and fat in the mitochondrial matrix, as well as providing the building blocks for fatty acid synthesis in the cytosol (Pietrocola et al., 2015). However, these metabolites also impact cellular function by *N*-acylating the ε-amino of protein lysines. The significance of *N*-acylation is implied by the existence of several sirtuins (Sirt1–7), which use NAD^+^ to remove acetyl and other acyl groups from protein lysines and are important in the pathology of a wide range of degenerative diseases, including cancer, aging, and diabetes (McDonnell et al., 2015, Pan and Finkel, 2017). *N-*acetylation was originally considered to be solely a regulatory modification, allowing the cell to respond to acetyl-CoA, the acetyl-CoA/CoA ratio, or NAD^+^. This was reassessed after observation of several thousand sites of lysine *N-*acetylation *in vivo* (Rardin et al., 2013, Weinert et al., 2015, Baeza et al., 2016), with the vast majority having a very low (∼0.1%) stoichiometry of acetylation (Weinert et al., 2015, Weinert et al., 2017, James et al., 2017). Lysines of mitochondrial proteins can be non-enzymatically *N*-acetylated *in vitro* by acetyl-CoA (Wagner and Payne, 2013, James et al., 2017), and other acyl-CoAs, such as succinyl-CoA, malonyl-CoA, and glutaryl-CoA, also generate *N-*linked modifications on lysines *in vivo* without known transferase enzymes (Weinert et al., 2013, Peng et al., 2011, Tan et al., 2014). Consequently, it was proposed that acyl-CoAs represent a “carbon stress,” through which chronic exposure to acyl-CoAs causes cumulative cellular damage that contributes to degenerative diseases and aging, as well as explaining the benefits of sirtuins and dietary restriction (Wagner and Hirschey, 2014, Trub and Hirschey, 2018, Weinert et al., 2017).

Non-enzymatic *N-*acylation occurs when the amine group (pK_a_ ∼10.5) of a protein lysine deprotonates to become a nucleophile, which then attacks the thioester carbonyl of an acyl-CoA to generate a stable amide-linked modification (Wagner and Payne, 2013). However, lysines are not the only nucleophilic residues that can react with acyl-CoAs (Bizzozero et al., 2001, James et al., 2017). The deprotonated thiol of a cysteine (pK_a_ ∼8.5) can be non-enzymatically *S-*acylated, and *in vitro* cysteine *S-*acetylation by acetyl-CoA is ∼100-fold more rapid than the corresponding *N-*acetylation of a lysine (James et al., 2017). However, although cysteine reactivity is greater, the thioester bond of an *S-*acyl cysteine is less stable and prone to further nucleophilic attack. In this way, *S*-acylated cysteines can cause *N*-acylation of nearby lysines via subsequent *S→N* transfer of their acyl moiety (James et al., 2017, Cohen et al., 2013). The progression of this *S→S→N*-acyl transfer reaction has been shown *in vitro* on a synthetic peptide and on mitochondrial membrane proteins (James et al., 2017). Furthermore, protein *N-*acetylation is diminished by cysteine alkylation (James et al., 2017), cysteine mutation prevents auto-catalytic lysine *N-*acetylation of Tau protein (Cohen et al., 2013), and sites of lysine *N-*acetylation and cysteine *S-*acylation are often observed on the same peptide in mouse liver (Rardin et al., 2013, Gould et al., 2015, James et al., 2017). There are also rapid enzymatic and non-enzymatic examples of similar *S→S→N* transfer reactions, where the second rate-limiting step is enhanced by proximity, including native chemical ligation and ubiquitin ligation.

Although examples of this chemistry exist, the extent that surface cysteines generally enhance lysine *N-*acetylation *in vivo*, in the presence of pathways that might mitigate against it, is unclear. Here, we explore whether surface cysteines enhance lysine *N-*acetylation *in vivo*, using an existing mouse liver proteomic dataset that reports the stoichiometry of lysine *N*-acetylation at each site (Weinert et al., 2015). From these *N*-acetylated peptides and the crystal structures of homologous proteins, we generated mouse structural models of 619 proteins that are *N-*acetylated *in vivo* and show that lysine *N*-acetylation is increased by proximity to a surface cysteine. Furthermore, we found decreased conservation of pairs of proximal cysteine and *N-*acetylated lysines (CysLys) in the genomes of 52 other mammal and bird species. The low conservation of *N-*acetylated CysLys pairs strongly correlated with maximal lifespan, and this was not observed for proximal pairs of serine and *N-*acetylated lysines (SerLys). Our results support a model where lysine *N*-acetylation is a non-enzymatic byproduct of high concentrations of acetyl-CoA, with effects that favor decreased conservation of *N-*acetylated CysLys pairs in long-lived species.

## Results

### Generating a 3D Structural Library of Mouse Liver Proteins with *N*-Acetylated Lysines

**Figure 1:**
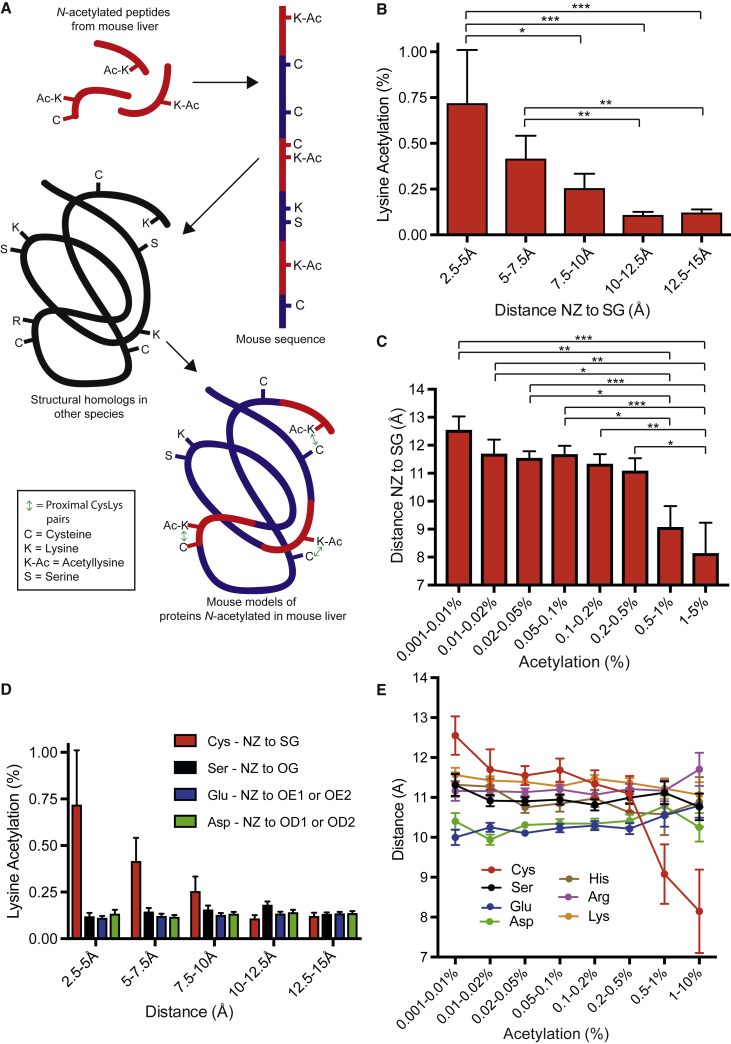
Protein Lysine *N-*Acetylation Is Increased by Proximity to a Cysteine (A) Creation of structural library of proteins with *N-*acetylated lysines in mouse liver *in vivo*. The set of 4,320 *N-*acetylated peptides is from Weinert et al. (2015). Mouse structural models of 619 proteins with *N-*acetylated lysines were generated based on molecular structures in other species, and the distance between their lysine amine (NZ) and cysteine thiol (SG) atoms was calculated. (B) Lysine *N-*acetylation increases with proximity to a cysteine thiol. All *N-*acetylated CysLys pairs <15 Å apart were grouped by NZ to SG distance. (C) The most *N-*acetylated lysines are closer to cysteine thiols. All *N-*acetylated CysLys pairs <15 Å apart were grouped by their degree of *N-*acetylation. (D) Lysine *N-*acetylation is not caused by serines, glutamates, or aspartates. Pairs <15 Å apart were grouped by their NZ to SG, OG, OE1/OE2, or OD1/OD2 distances. (E) The most *N-*acetylated lysines are not closer to serines, glutamates, aspartates, histidines, arginines, or other lysines. Pairs <15 Å apart were grouped by their degree of *N-*acetylation. Data are the mean ± SEM. ^∗^p < 0.05; ^∗∗^p < 0.01; ^∗∗∗^p < 0.001; ns, not significant.

Primary protein sequences are often searched for motifs that increase reactivity of certain residues *in vivo*, but the proximity of two residues cannot consistently be predicted like this. As molecular structures for many proteins now exist, we created a dataset of distances between *N-*acetylated lysines and nearby surface cysteines to see whether cysteines influence lysine *N-*acetylation *in vivo* (Figure 1A).

We began with a stoichiometric dataset that identified 4,320 *N-*acetylated lysines in mouse liver (Weinert et al., 2015, Baeza et al., 2016). We took the protein sequences of the *N-*acetylated peptides from this and built single-subunit models of the mouse proteins using the most homologous existing structure as a template (Fiser and Sali, 2003). Modeling was required as *N-*acetylated lysines, and their proximal cysteines were often absent from homologous structures in other species. For each model, we calculated distances from the amine nitrogen atom (NZ) of all lysines to the thiol sulfur atom (SG) of all cysteines. This created 147,880 NZ to SG distances on 619 proteins that could be correlated with the initial stoichiometric acetylation dataset (Weinert et al., 2015). As only surface residues can be *N-*acetylated, we considered only pairs of cysteine and lysine (CysLys) residues where both the NZ or SG atoms were solvent accessible (>5 Å^2^) in the modeled single subunit. This 5 Å^2^ cutoff is ∼7% of their potential solvent accessible surface area, as the maximum observed for NZ and SG atoms was 64.7 Å^2^ and 74.5 Å^2^, respectively (Table S1). Although 88.8% of lysine amines were exposed, 61.2% of cysteine thiols were buried internally. This left 52,321 CysLys pairs in which both the NZ and SG atoms were on the surface of the modeled subunit.

The 619 proteins have an average solvent-accessible surface area of 20,862 Å^2^, corresponding to a sphere with a radius of ∼40 Å. At this size, interaction between two surface residues more than ∼15 Å apart is often blocked by intervening surface features or by curvature of the protein surface (Figure S1A). Thus, analysis was limited to 2,595 CysLys pairs where NZ and SG atoms were <15 Å apart (Table S1). Of these pairs, 376 were *N-*acetylated on their lysine to some degree (average acetylation = 0.19%). Supporting our structural approach for finding motifs, only 124 of the 376 (33%) acetylated CysLys pairs that were <15 Å apart also lay within 10 residues on the primary sequence (median = 27 residues separation; Table S1).

Of the 376 *N-*acetylated CysLys pairs, 48 of the cysteines (12.7%) had previously been identified as *S-*acylated (Gould et al., 2015). This is significantly higher than non-*N*-acetylated pairs, where only 118 of the remaining 2,219 (5.3%) have an *S-*acylated cysteine (p < 0.0001; Table S1). This is consistent with *S→S-*acyl exchange from acetyl-CoA to a protein cysteine followed by *S→N-*acyl transfer to a nearby lysine.

### Close Proximity of a Cysteine Enhances Lysine *N*-Acetylation *In Vivo*

One prediction of our model is that the closer cysteines and lysines are, the greater the extent of lysine acetylation. When the 376 CysLys pairs <15 Å apart (Table S1) were grouped by their NZ to SG distances, there was a significant increase in lysine *N-*acetylation when a cysteine was <7.5 Å away, and this declined with distance (Figure 1B). As each surface lysine is paired with every surface cysteine on the protein, the prior analysis filtered out distant CysLys pairs (>15 Å). To show Figure 1B was not an artifact of filtering, a further dataset was created with only the distance from each lysine to the nearest surface cysteine (Table S1; Figure S1B). When these 2,387 CysLys pairs were grouped by NZ to SG distances, there was again a significant increase in lysine *N-*acetylation when CysLys pairs were <7.5 Å apart. Finally, to confirm the result was independent of how the dataset was grouped, the initial 376 CysLys pairs were grouped by their degree of *N-*acetylation (Table S1). The two most *N-*acetylated groups (>0.5%) were significantly closer than the ∼11.5 Å separation observed with less acetylated CysLys pairs (Figure 1C). Plotting individual CysLys pairs also showed a significant correlation between lysine *N-*acetylation and NZ to SG distance (Figure S1C). In summary, cysteines enhance lysine *N-*acetylation *in vivo*, and this decays to background when separation is >10–11.5 Å (Figures 1B, 1C, S1B, and S1C).

### Proximity to Other Charged Residues Does Not Enhance Lysine *N*-Acetylation *In Vivo*

To show it is the reactive thiol of a cysteine that promotes lysine *N-*acetylation, we assessed the effect of serines on lysine *N-*acetylation. Serines are structurally identical to cysteines, except the reactive sulfur (SG) atom of cysteine is an oxygen atom (OG) that will not lead to lysine *N-*acetylation. As expected, the NZ to OG distance did not correlate with lysine *N-*acetylation, consistent with thiol reactivity leading to lysine *N-*acetylation (Table S1; Figures 1D, 1E, and S1D). In the active sites of enzymes, charged amino acids can enhance the reactivity of nucleophiles by stabilizing their deprotonated form. However, proximity of a lysine to the acidic moieties of glutamate or aspartate, or to the basic groups of histidine, arginine, and lysine, showed no correlation with lysine *N-*acetylation (Table S1; Figures 1D, 1E, and S1E–S1I).

### Most Proximal *N*-Acetylated CysLys Pairs Are Less Conserved

**Figure 2:**
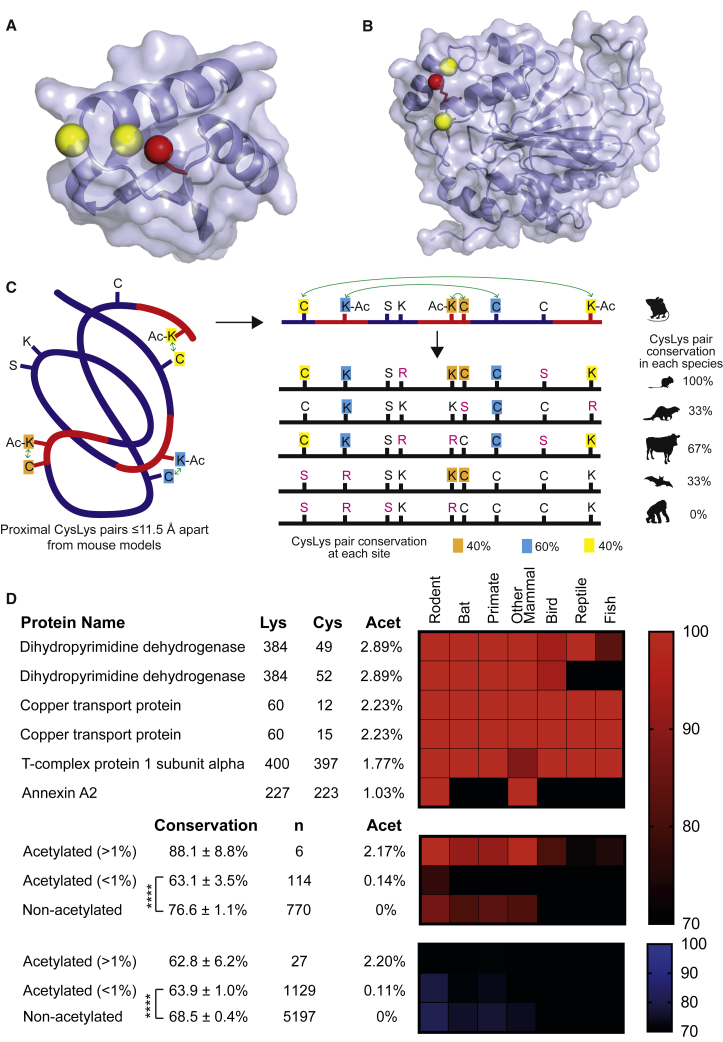
Most Proximal *N-*Acetylated CysLys Pairs Are Not Conserved (A) A mouse model of human persulfide dioxygenase (4CHL) contains a lysine amine (red) that is *N-*acetylated flanked by two cysteine thiols (yellow). (B) A mouse model of human copper transport protein (3IWL) contains a lysine amine (red) that is *N-*acetylated near two cysteine thiols (yellow). (C) Schematic diagram for determining the degree to which proximal (≤11.5 Å) CysLys pairs are conserved. A CysLys pair is conserved only if both residues were present. Conservation of CysLys pairs at specific sites in a range of species is depicted vertically and conservation at a range of sites in a particular species is shown horizontally. (D) A few *N-*acetylated sites are conserved, but most are not. A heatmap of genomic conservation of individual CysLys (red) pairs *N-*acetylated by >1% in mouse in 66 vertebrate species. These have been grouped and compared to less acetylated CysLys pairs (<1%) and non-acetylated CysLys pairs. A heatmap of genomic conservation of SerLys pairs (blue) is shown. ^∗∗∗∗^p < 0.0001.

Three of the four most *N-*acetylated CysLys pairs <11.5 Å apart in the CysLys dataset have a second proximal cysteine <11.5 Å away (Table S1; Figures 2A, 2B, S2A, and S2B). Although the presence of multiple cysteines in close proximity could suggest *N-*acetylation of these lysines is functional, their low stoichiometry of *N-*acetylation *in vivo* argues otherwise. This illustrates an unresolved dichotomy in the field: the extent to which lysine *N-*acetylation is a regulatory modification or an unintentional byproduct of high *in vivo* concentrations of acetyl-CoA (Wagner and Hirschey, 2014, Trub and Hirschey, 2018).

One way to investigate this dichotomy, while incorporating the low *N-*acetylation stoichiometry present *in vivo* across a large range of acetylation sites, is to evaluate the conservation of CysLys pairs in diverse genomes (Figure 2C). CysLys pairs where regulatory modifications occur should be conserved, and sites of non-functional or detrimental acylation should be neutral or comparatively less conserved. For this, *N-*acetylated mouse proteins were aligned to their orthologs in 66 further vertebrate species (13 rodents, 6 bats, 8 primates, 9 other mammalian species, 16 birds, 7 reptiles, and 7 fish), and each of the 890 CysLys pairs was considered conserved only if both the cysteine and lysine were present in the ortholog (Table S2). Individually, the most acetylated (>1%) proximal CysLys pairs with identifiable orthologs were conserved within vertebrates (Figure 2D). This small group was more conserved across all 66 vertebrate species (88.1% ± 8.8%; n = 6), although not significantly so, than either the 114 weakly acetylated (<1%) proximal CysLys pairs (63.1% ± 3.5%), the 770 proximal CysLys pairs not observed as acetylated by mass spectrometry (MS) (non-acetylated; 76.6% ± 1.1%), or the 27 highly acetylated (>1%) proximal SerLys pairs (62.8% ± 6.2%). Strikingly, the weakly *N-*acetylated (<1%) group that contains 95% of proximal *N-*acetylated CysLys pairs was significantly less conserved than the non-acetylated proximal CysLys pairs (Figure 2D; p < 0.0001).

Thus, although selection of proximal cysteines to enhance lysine *N-*acetylation may occur in a few places, for the vast majority of sites, lysine *N-*acetylation at best serves no purpose and at worst negatively impacts the organism.

### Increased *N*-Acetylation in Matrix Leads to Compensatory Changes to CysLys Pairs

**Figure 3:**
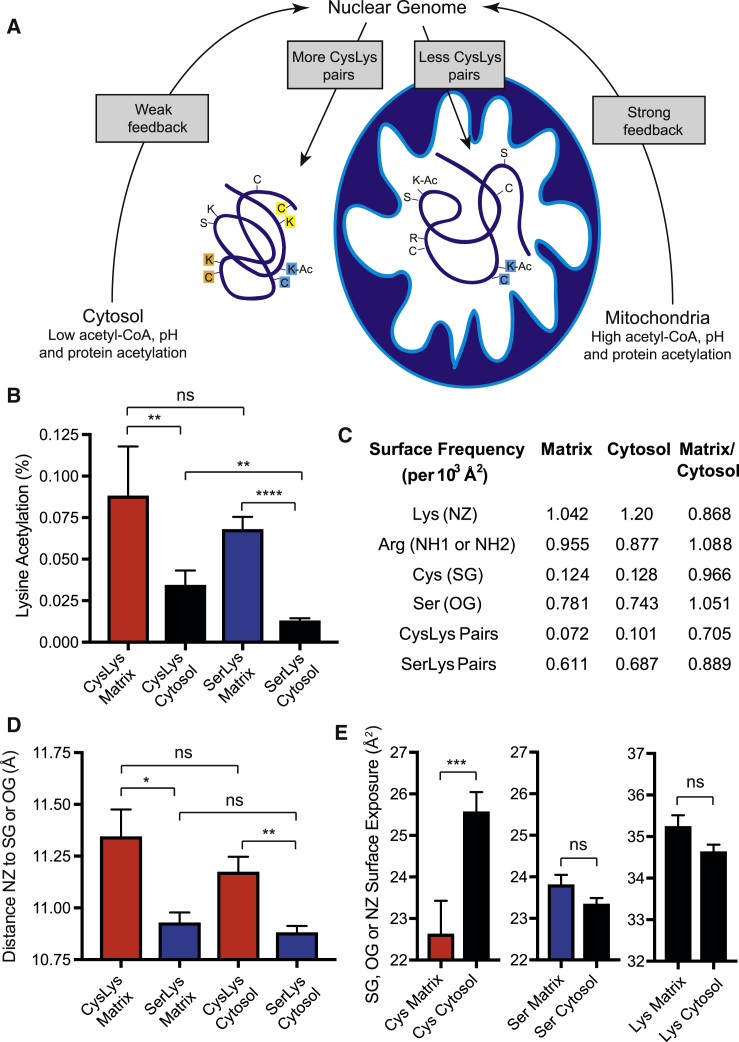
Loss of Proximal CysLys Pairs from the Mitochondrial Matrix (A) CysLys pairs may be lost from matrix proteins. (B) The mitochondria are a more acetylating environment than the cytosol. All CysLys and SerLys pairs <15 Å apart were segregated by cellular location. (C) Surface lysine and cysteines and CysLys pairs are less frequent in the matrix. (D) When present, CysLys pairs are further apart than SerLys pairs. All CysLys and SerLys pairs <15 Å apart were segregated by cellular location. (E) The cysteine of CysLys pairs is less solvent exposed in the matrix. SG and OG atom surface exposure is from CysLys and SerLys pairs, and NZ surface exposure is from both CysLys and SerLys pairs. Data are the mean ± SEM. ^∗^p < 0.05; ^∗∗^p < 0.01; ^∗∗∗^p < 0.001; ^∗∗∗∗^p < 0.0001.

The higher acetyl-CoA concentration and pH of the mitochondrial matrix make lysines in that compartment more susceptible to acetylation than those in the cytosol (Figure 3A) (Wagner and Payne, 2013, James et al., 2017). This effect was noted in the original study (Weinert et al., 2015), and our re-analysis shows 20.1% (41/204) of proximal CysLys pairs are *N-*acetylated in the matrix compared to 13.4% of proximal CysLys pairs (90/669) in the cytosol (p = 0.02). Furthermore, for mitochondrial proteins, the average *N-*acetylation of CysLys pairs and SerLys pairs is 2.6-fold and 5.2-fold higher, respectively, than those in the cytosol (Figure 3B).

If most low-level lysine *N-*acetylation negatively impacts organismal fitness, then adaptation of cytosolic and mitochondrial proteins in response to increased *N-*acetylation may have occurred (Figure 3A). Consistent with this, the surface density of exposed lysine (NZ) and cysteine (SG) atoms was 13.2% and 3.4% lower, respectively, in the mitochondrial matrix relative to the cytosol (Figure 3C; Table S3). These changes were matched by 8.8% and 5.1% increases in the surface density of less reactive arginine (NH1/NH2) and serine (OG) atoms of matrix proteins. These differences were reflected in a 29.5% decrease in the surface density of potentially reactive (<11.5 Å) CysLys pairs in the matrix. Where proximal CysLys pairs exist, they are significantly further apart by 0.42 Å and 0.29 Å than corresponding SerLys pairs in the matrix (p = 0.015) and cytosol (p = 0.001), respectively (Figure 3D; see also Figure 1E). This may prevent some interactions and, where interaction is possible, this ∼3% increase in distance (Å) may lower the *S-*acetyl cysteine concentration (Å^−3^) near lysines by ∼10%. Finally, solvent exposure of the SG atom of proximal CysLys pairs was 11.5% lower in the matrix than in the cytosol (p = 0.0008; Figure 3E).

This is consistent with increased lysine *N-*acylation in the matrix causing compensatory changes in mitochondrial protein to limit lysine *N-*acylation.

### *N*-Acetylated Cytosolic CysLys Pairs Are Also Less Conserved

**Figure 4:**
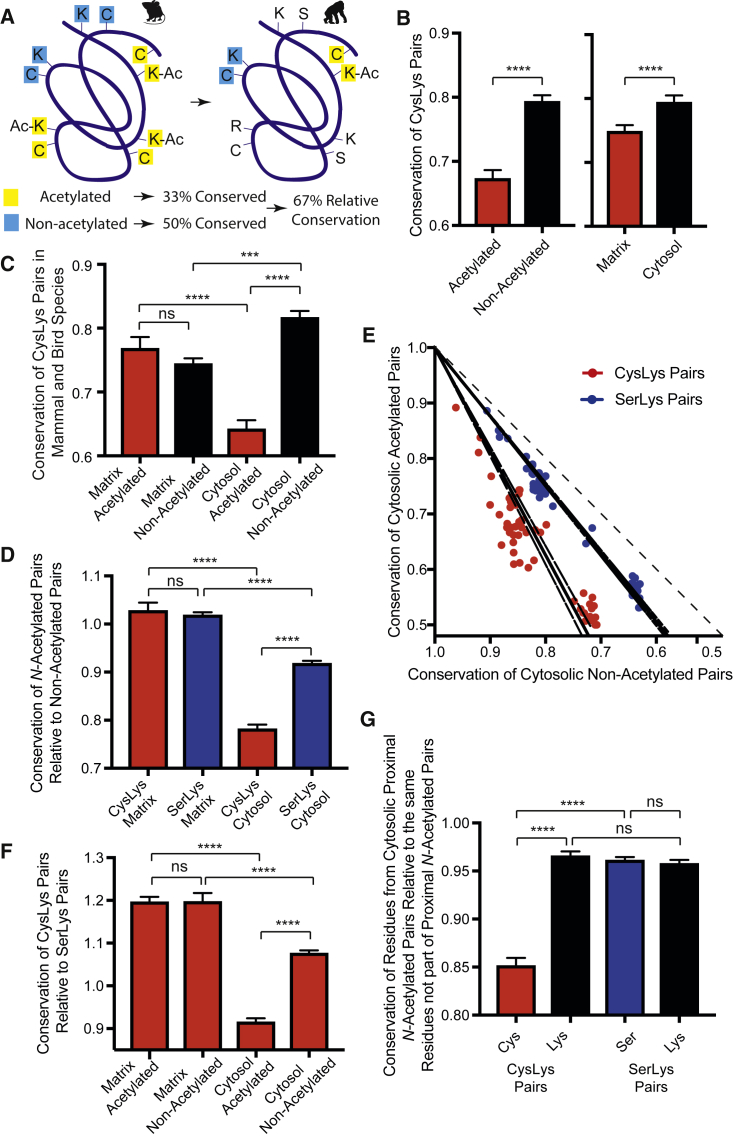
Less Conservation of Proximal *N-*Acetylated CysLys Pairs in 52 Other Mammals and Bird Species (A) Example of pair conservation. (B) CysLys pairs are less conserved if they are *N-*acetylated or located in the matrix. (C) Conservation of cytosolic *N-*acetylated CysLys pairs is less than other CysLys pairs. (D) *N-*acetylated CysLys pairs are less conserved than non-acetylated CysLys pairs in the cytosol. Data are the average conservation of *N-*acetylated pairs relative to non-acetylated pairs per species. (E) Cytosolic *N-*acetylated CysLys pairs are less conserved than non-acetylated CysLys pairs. Data are the conservation of *N-*acetylated pairs relative to non-acetylated pairs in each species with linear regression lines and 95% confidence intervals. The line at 45° indicates where values should lie if conservation of *N-*acetylated and non-acetylated pairs was similar. (F) Cytosolic *N-*acetylated CysLys pairs are less conserved than SerLys pairs. Data are average conservation of *N-*acetylated CysLys pair relative to *N-*acetylated SerLys pairs. (G) Cytosolic cysteines from *N-*acetylated CysLys pairs are less conserved than other surface cysteines. Data are the average cysteine, lysine, or serine conservation from cytosolic *N-*acetylated pairs relative to all other cytosolic surface cysteines, lysines, or serines not part of these *N*-acetylated pairs. Data are the mean ± SEM. ^∗∗∗^p < 0.001; ^∗∗∗∗^p < 0.0001.

The above comparison cannot explore whether proximal cytosolic *N-*acetylated CysLys pairs are also detrimental, so we next considered conservation of CysLys pairs in each species (Figure 4A; see also Figure 2C). This analysis was kept to 36 mammalian (13 rodent, 6 bat, 8 primate, and 9 other mammalian species) and 16 bird species whose body temperature is similar as non-enzymatic acetylation is temperature dependent (James et al., 2017). Within species, CysLys pairs observed as *N-*acetylated in mouse were less conserved than CysLys pairs not observed as acetylated by MS (Figure 4B), and CysLys pairs of matrix proteins were less conserved than CysLys pairs of cytosolic proteins (Figure 4B). When Figure 4B is further partitioned, *N-*acetylation of cytosolic proteins is also detrimental (Figure 4C).

Genetic distance from mouse is a confounding variable, so conservation of proximal *N-*acetylated CysLys pairs (≤11.5 Å) was expressed relative to that of proximal CysLys pairs where the lysine was not observed as *N-*acetylated by MS (Figure 4A). If *N-*acetylated and non-acetylated pairs experience similar selective pressures, they will evolve equally and their relative conservation values will be ∼1. Differences between *N-*acetylated and non-acetylated CysLys and SerLys pairs do not occur for mitochondrial matrix proteins, as their relative conservation values are ∼1 (Figure 4D). In contrast, acetylated CysLys pairs were less conserved than non-acetylated CysLys pairs on cytosolic proteins (p = 5.5 × 10^−29^; Figure 4D). The basis for this result can be seen in a plot of the individual species, where the conservation rate deviates from 45° with all 52 species lying below this line (Figure 4E). This relative conservation rate for cytosolic CysLys pairs was lower than for SerLys pairs (Figures 4D and 4E). This equates to greater mutation of *N-*acetylated cytosolic CysLys pairs (87.1% ± 4.1%) and SerLys pairs (23.5% ± 1.0%) than their non-acetylated counterparts (Figure 4D). Individual cysteines are often functional, and protein cysteines as a group form a large redox buffer (Requejo et al., 2010). Consistent with this, cytosolic non-acetylated as well as matrix *N-*acetylated and non-acetylated CysLys pairs are all more conserved than corresponding SerLys pairs (Figure 4F). In contrast, conservation of cytosolic *N-*acetylated CysLys pairs relative to cytosolic *N-*acetylated SerLys pairs is less than one (Figure 4F). This does not arise from differences in the mutation rate between mitochondrial and nuclear genomes, as all analyzed proteins are encoded by the nuclear genome.

This suggests, within vertebrates, cytosolic CysLys pairs are tolerated unless they catalyze *S→N*-acyl transfer reactions. In contrast, the acetylating environment of the matrix may have meant readily mutable lysines and CysLys pairs were early targets during the evolution of eukaryotes (Figure 3), and change is difficult to observe within vertebrates (Figure 4).

### Cysteines of *N*-Acetylated Cytosolic CysLys Pairs Are Less Conserved

These differences could arise from mutation of either the cysteine, the lysine, or both residues of an *N-*acetylated CysLys pair. Thus, conservation of each cysteine and lysine of a proximal *N-*acetylated CysLys pair (≤11.5Å) was considered independently (Table S4; Figure 4G). Cysteines that were part of proximal *N-*acetylated CysLys pairs in the cytosol were less conserved than cytosolic cysteines not part of proximal *N-*acetylated CysLys pairs. This was not observed for lysines that were part of proximal *N-*acetylated CysLys pairs in the cytosol. It was also not observed for serines or lysines that were part of proximal *N-*acetylated SerLys pairs. As these groups were determined by lysine *N-*acetylation, decreased cysteine conservation provides extra support for an interaction between proximal cysteines and lysines *in vivo*.

### Conservation of *N*-Acetylated CysLys Pairs on Cytosolic Proteins Inversely Correlates with Lifespan

**Figure 5:**
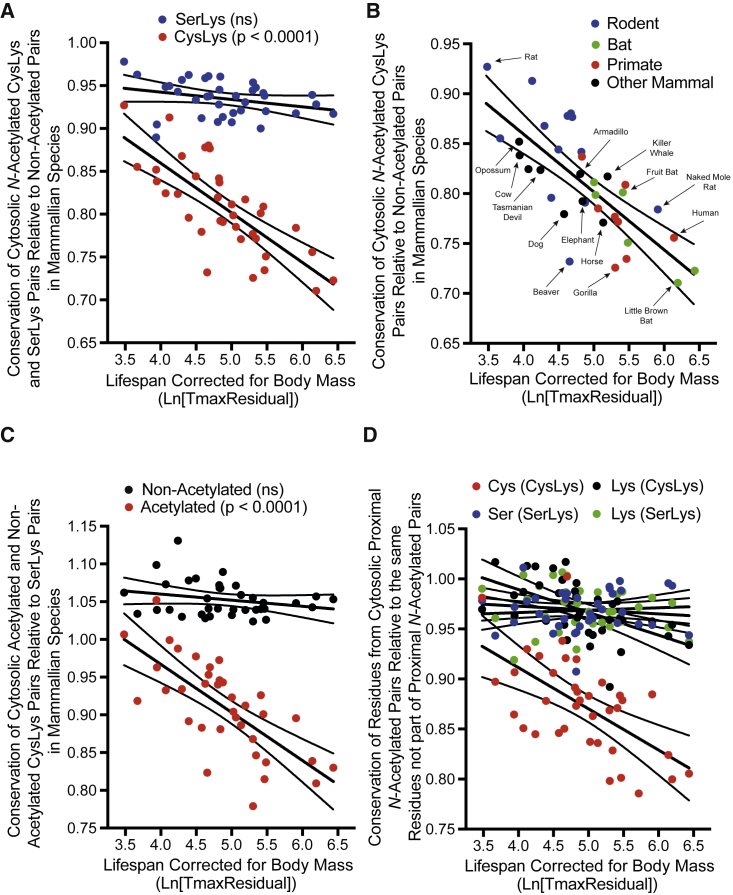
Lifespan Negatively Correlates with Conservation of Proximal Cytosolic *N-*Acetylated CysLys Pairs in 36 Other Mammalian Species T_max_Residual is the maximum lifespan of a species as a fraction of the maximum lifespan expected for a mammal of its body mass. (A) Cytosolic *N-*acetylated CysLys pair conservation negatively correlates with T_max_Residual in mammals. Data are the conservation of cytosolic *N-*acetylated CysLys (red) and SerLys (blue) pairs relative to non-acetylated pairs. (B) Phylogeny does not explain the correlation between cytosolic *N-*acetylated CysLys pair conservation and T_max_Residual. Data are *N-*acetylated CysLys pair conservation in rodents (blue), bats (green), primates (red), and other mammals (black) relative to non-acetylated pairs. Common names are indicated with scientific names in Table S2. (C) Conservation of cytosolic *N-*acetylated CysLys pairs when expressed relative to *N-*acetylated SerLys pairs negatively correlates with T_max_Residual. Data are conservation of *N-*acetylated (red) and non-acetylated (black) CysLys pairs relative to SerLys pairs. (D) Conservation of cytosolic cysteine and lysines that are part of *N-*acetylated proximal CysLys pairs negatively correlates with T_max_Residual. Proximal cytosolic *N-*acetylated pairs were separated into constituent residues, and conservation of cysteines (red) and lysines (black) part of *N-*acetylated CysLys pairs and serines (blue) and lysines (green) part of *N-*acetylated SerLys pairs was considered independently relative to all other cysteines, serines, or lysines in the cytosol. Lines of best fit are linear regression lines with 95% confidence intervals.

Decreased conservation of proximal *N-*acetylated CysLys pairs suggests that lysine *N-*acetylation is detrimental. As dietary restriction and enzymes that could affect cytosolic lysine *N-*acylation are associated with changes in lifespan (Kanfi et al., 2012, Lin et al., 2000, Peleg et al., 2016), we hypothesized that longer-lived animals might favor the loss of proximal CysLys pairs if the lysine can be *N-*acetylated. When conservation of *N-*acetylated CysLys pairs relative to non-acetylated CysLys pairs was plotted against the maximum lifespan of 52 mammal and bird species, there was a highly significant negative correlation (p < 0.0001) and no significant correlation for *N-*acetylated cytosolic SerLys pairs (p = 0.32; Figure S3A). Body mass is a confounding variable when using maximum lifespan, because larger animals generally live longer (Tacutu et al., 2013, Ma and Gladyshev, 2017). Despite no correlation of weight with *N-*acetylated CysLys pair conservation (p = 0.96; Figure S3B), it was desirable to investigate longevity independently of body mass. To correct for body mass, we used T_max_Residual, which is the observed maximum lifespan of a species as a fraction of the maximum lifespan expected for a mammal of that body mass (Tacutu et al., 2013, Ma and Gladyshev, 2017). When ln(T_max_Residual) is plotted against cytosolic *N-*acetylated CysLys pair conservation relative to cytosolic non*-*acetylated CysLys pair conservation, a highly significant negative correlation is evident (p < 0.0001; Figure S3C), and this is diminished for cytosolic *N-*acetylated SerLys pairs (p = 0.014; Figure S3C). T_max_Residual was developed for mammals, and the correlation is even clearer if just mammals are considered (Figure 5A). This trend is not significant for SerLys pairs in the cytosol (Figure 5A) or for CysLys and SerLys pairs in the mitochondrial matrix (Figure S3D). Nor is it easily explained by phylogeny as mouse clusters with short-lived marsupials from which it diverged ∼160 million years ago, yet is distant from long-lived bats and primates with which it last shared ancestors ∼80–100 million years ago (Figures 5B and S3E). This correlation is not caused by clustering of species closely related to mouse as it remains if rodents are excluded and just distant mammalian relatives are considered (Figure S3E). Furthermore, individual mammalian orders show similar correlations (Figure S3F), and the correlation in Figure 5A remains highly significant (p = 0.0009) after correction with phylogenetic generalized least-squares (PGLSs) (Figure S4; Table S5).

So far, genomic divergence of *N-*acetylated CysLys pairs has been controlled for by comparing it with conservation of CysLys pairs on the surface of the modeled subunits, where no *N-*acetylated peptide was observed by MS. However, there are caveats to this, as non-acetylated pairs could be *N-*acetylated and not detected or buried within the quaternary structure *in vivo*. Thus, genomic divergence was also controlled for by expressing conservation of *N-*acetylated CysLys pairs relative to *N-*acetylated SerLys pairs so all lysines are exposed to acetyl-CoA *in vivo* and have been experimentally observed as *N-*acetylated by MS (Weinert et al., 2015). Again, conservation of cytosolic *N-*acetylated CysLys pairs is lower in species with a longer than expected lifespan, and this correlation is highly significant (Figure 5C; p < 0.0001). This correlation is not observed with non-acetylated CysLys pairs (Figure 5C) or matrix *N-*acetylated CysLys pairs (Figure S5A), and it is not explained by phylogeny (Figures S5B–S5D). The correlation in Figure 5C remains highly significant after PGLS (p = 0.0019; Figure S4; Table S5). Thus, the trend arises from *N*-acetylated CysLys pairs and not the procedure used to control for genomic divergence.

Finally, we assessed whether the correlation of conservation of proximal acetylated CysLys pairs in the cytosol with lifespan was caused by their cysteine or lysines (Figure 5D). There was a significant correlation between ln(T_max_Residual) and conservation of cysteines that were part of proximal *N-*acetylated CysLys pairs relative to all other cysteines (p = 0.0002). This correlation was also significant for lysines from proximal *N-*acetylated CysLys pairs (p = 0.0005) but absent from serines and lysines from proximal *N-*acetylated SerLys pairs (Figure 5D). The correlations in Figure 5D remain significant after PGLS (Figure S4; Table S5).

In summary, both lifespan and lifespan corrected for body mass correlate with the conservation of proximal *N-*acetylated cytosolic CysLys pairs. This is not observed with SerLys pairs, non-acetylated CysLys pairs, or CysLys pairs in the mitochondrial matrix.

## Discussion

Lysine *N-*acetylation by acetyl-CoA can be prevented by changing a proximal cysteine to a serine on a peptide or protein (James et al., 2017, Cohen et al., 2013). By generating a 3D structural dataset of mouse proteins previously observed to be *N-*acetylated in mouse liver (Weinert et al., 2015), we show the degree of lysine *N-*acetylation correlates with proximity to a cysteine *in vivo* (Figure 1). That migration of acyl groups from cysteine to proximal lysines occurs *in vivo* is further supported by genomic adaptations between cellular compartments (Figures 3 and 4). In particular, cytosolic proximal *N-*acetylated CysLys pairs were less conserved (Figures 2D and 4), and this primarily resulted from lower cysteine conservation (Figure 4G). As CysLys pairs were grouped by lysine *N-*acetylation, cysteine evolution and lysine *N-*acylation are dependent events. Together, these results indicate that *S→N* transfer reactions are a feature of protein surfaces *in vivo*. Although CysLys pairs were identified using an acetylation dataset, other acyl-CoAs would react with the same pairs, as might reactive dicarbonyls, such as methylglyoxal (Schwarzenbolz et al., 2008).

Average acetylation of lysines with cysteines ≤11.5 Å away is 86% higher (p = 0.0002) than other *N-*acetylated lysines. Consequently, each of these lysines contributes disproportionately to the *N-*acetylation load *in vivo*. However, only 5.7% of *N-*acetylated lysines have an intramolecular cysteine ≤11.5 Å away. Thus, we estimate proximal cysteines account for 10.7% of the total *N-*acetylation load that occurs on liver proteins *in vivo*, with other mechanisms, such as association of acetyl-CoA with the surface of proteins (Tsuchiya et al., 2017), intermolecular *S→S→N-*acetyl transfer reactions, and direct *N-*acetylation, also contributing to acetylation load. The relative importance of each of these mechanisms will differ in each cellular compartment and tissue. For example, only the *S→S→N-*acyl transfer reaction is sensitive to glutathione (GSH) and hydroxyacyl glutathione hydrolase (HAGH) and liver has a particularly high GSH concentration relative to other tissues (James et al., 2017). As the proteomic study used here is from liver and not a post-mitotic tissue often associated with aging, this may also explain the small contribution of the *S→S→N-*acetyl transfer reaction to *N-*acetylation load despite the strong correlation of proximal *N-*acetylated CysLys pair conservation with lifespan. Even so, our results imply low stoichiometry lysine *N-*acetylation across a range of sites has a functional impact large enough to affect genome-wide changes (Figures 3 and 4).

Exactly why sites of lysine *N-*acetylation are less conserved is beyond the scope of this paper. However, the lack of conservation of a large number of CysLys pairs and the generally low stoichiometry suggest the impact of lysine *N-*acylation is cumulative across a range of sites (Wagner and Hirschey, 2014, Weinert et al., 2015, Trub and Hirschey, 2018). This is supported by recent work showing sirtuin deacetylases suppress acetylation below baseline at a range of sites (Weinert et al., 2017). One potential mechanism is aberrant proteostasis, as autophagy is often required for lifespan extension (Nakamura and Yoshimori, 2018) and lysine *N-*acylation removes ubiquitination sites and positive charges from the surface of a protein, which could increase protein aggregation (Kuczyńska-Wiśnik et al., 2016). Supporting this, *N-*acetyllysine-binding bromodomain proteins are involved in protein aggregation (Olzscha et al., 2017); *N-*acetylated inclusion bodies are larger and harder to resolve (Kuczyńska-Wiśnik et al., 2016); tracts of glutamine, an *N-*acetyl lysine mimetic, lead to aggregation of proteins, such as Huntingtin; and *N-*acetylation of a lysine via a cysteine leads to pathological aggregation of Tau protein (Cohen et al., 2011, Cohen et al., 2013).

Such aberrant proteostasis could also explain differences between compartments, as proteins in each are exposed to different acyl-CoAs and aldehydes and have distinct mechanisms of turnover (Green and Levine, 2014, Nakamura and Yoshimori, 2018). That it is conservation of cytosolic *N-*acetylated CysLys pairs that correlates with maximum lifespan (Figure 5) is consistent with genetic interventions that increase longevity. Sir2, a cytosolic deacetylase, is required for dietary restriction to extend lifespan in yeast (Lin et al., 2000), and its mammalian homolog, Sirt1, extends lifespan when overexpressed in the brain (Satoh et al., 2013). Overexpression of Sirt6, a cytosolic long-chain deacylase, extends lifespan in mice (Kanfi et al., 2012), as does knockdown of ATP-citrate lyase, which generates cytosolic acetyl-CoA and thus other acyl-CoAs in flies (Peleg et al., 2016). We note that maximum lifespan is the longest that any individual from a species has been recorded to live, e.g., 122.5 years for humans (Table S2), and it is likely this individual led a relatively healthy lifestyle. Thus, matrix CysLys pairs may be relevant in pathological settings, such as metabolic syndrome, where overnutrition has been linked to the mitochondrial isoform, Sirt3 (Hirschey et al., 2011, McDonnell et al., 2015).

In summary, we have created a 3D library of acetylated proteins from an existing dataset of mouse liver peptides containing *N-*acetylated lysines (Weinert et al., 2015). This library shows that the enhancement of lysine *N-*acetylation by proximal cysteines occurs at a range of sites *in vivo* (Figure 1). Furthermore, proximal CysLys pairs are less conserved if they can be *N-*acetylated (Figures 2, 3, and 4), and their degree of conservation on cytosolic proteins correlates with maximal lifespan in a large dataset of 52 species each with ∼500 proximal CysLys pairs (Figure 5). Which *N-*acyl lysine modifications exert the most selective pressure, which cellular processes and tissues are most affected, and whether lysine *N-*acyl modifications have a causative role in maximum lifespan or other degenerative pathologies remain to be elucidated.

## Experimental Procedures

### Creation of Mouse Structural Models

A list of *N-*acetylated peptides from mouse liver tissue (Weinert et al., 2015) was used to generate structural models of mouse proteins *N-*acetylated *in vivo*. This and other methodology is described in greater detail in Supplemental Information.

### Statistics and Data Processing

Statistical significance was determined using a two-tailed Student’s t test or one-way ANOVA followed by a Dunnett’s multiple comparison test. Differences in frequency were tested using two-sided chi-square tests. For linear regression, lines are displayed with 95% confidence intervals. Where indicated, p values were corrected for phylogenetic bias using the PGLS method with Pagel’s λ to estimate phylogenetic signal.

## Supplemental Information

Document S1. Supplemental Experimental Procedures and Figures S1–S5

Table S1. Proximity of Surface Residues, Related to Figure 1 Distance between reactive atoms on the surface of modeled proteins acetylated in mouse liver.

Table S2. Conservation of Surface Pairs, Related to Figures 2, 3, 4, and 5 Conservation CysLys or SerLys ≤ 11.5Å apart in mouse in the genomes of 66 other species.

Table S3. Frequency of Surface Residues, Related to Figure 3 Frequency of potentially reactive residues and pairs on the surface of acetylated proteins.

Table S4. Conservation of Residues from Surface Pairs, Related to Figures 4 and 5 Conservation of surface lysine, cysteine and serine residues from CysLys or SerLys pairs ≤ 11.5Å apart in mouse in the genomes of 66 other species.

Table S5. PGLS Correction, Related to Figure 5 P values adjusted for phylogeny.

Document S2. Article plus Supplemental Information
