# Supplementary material for: Proximal Cysteines that Enhance Lysine N-Acetylation of Cytosolic Proteins in Mice Are Less Conserved in Longer-Living Species
Source: Cell Rep. 2018 Aug 7;24(6):1445–55. doi: 10.1016/j.celrep.2018.07.007 (PMC6092265; doi:10.1016/j.celrep.2018.07.007)
Supplement: Document S1. Supplemental Experimental Procedures and Figures S1–S5 [file mmc1.pdf]

Cell Reports, Volume *24*

Supplemental Information

Proximal Cysteines that Enhance Lysine *N*-Acetylation of Cytosolic Proteins in Mice Are Less Conserved in Longer-Living Species

Andrew M. James, Anthony C. Smith, Cassandra L. Smith, Alan J. Robinson, and Michael P. Murphy

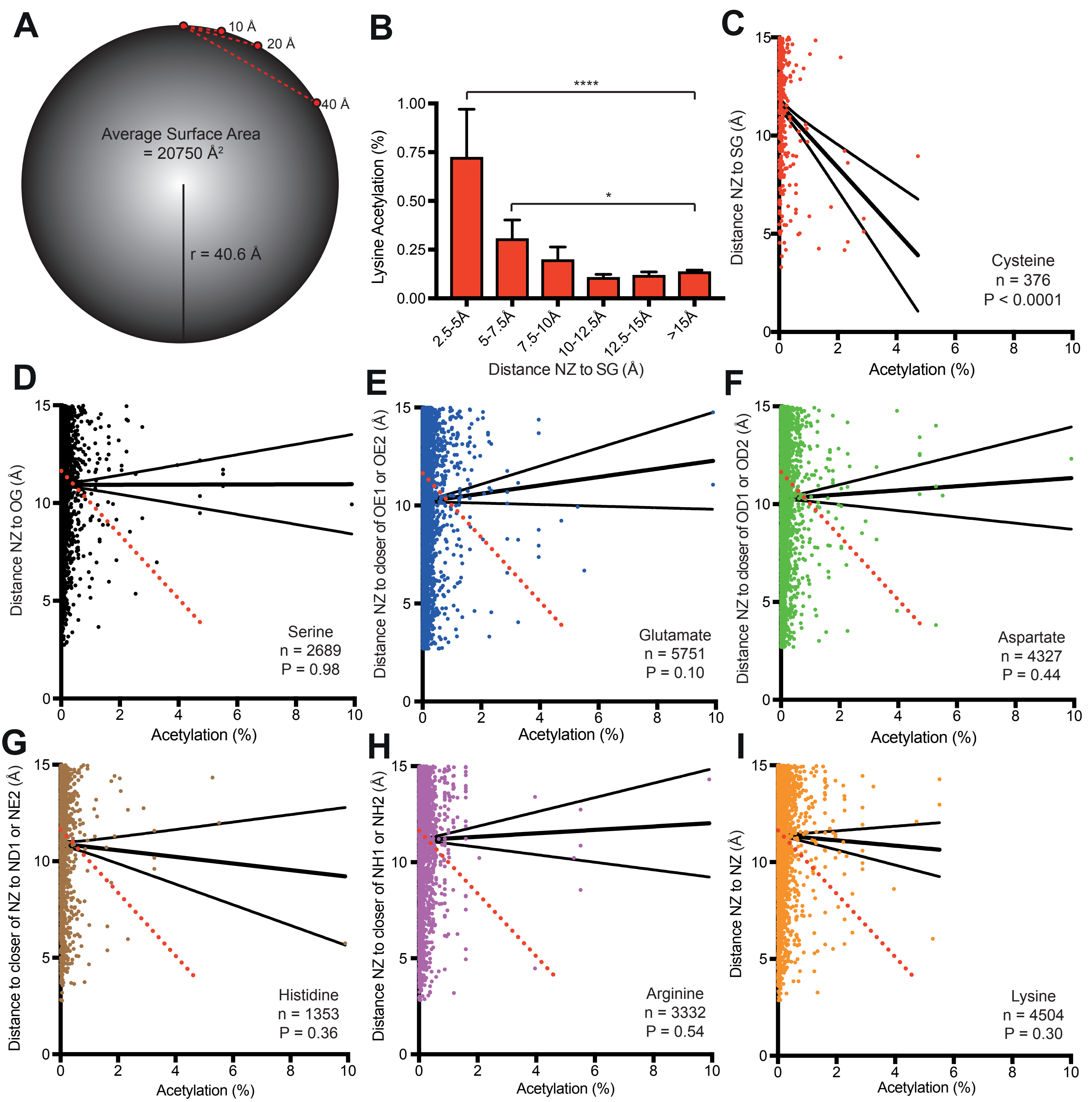

Figure S1. Protein lysine N-acetylation is increased by proximity to a cysteine. Related to Figure 1. The mouse liver dataset of acetylation sites and their degree of lysine N-acetylation is from (Weinert et al., 2015). Mouse homologs of 622 of these acetylated proteins were modelled from existing molecular structures in other species and the distance between their lysine amine (NZ) and a second atom was calculated using trigonometry. Only pairs where >5Å2 of both atoms were exposed on the surface, and where the NZ atom was N-acetylated, were included. A, reactions between residues >~15 Å apart are sterically hindered. The surface area of the 619 proteins was used to calculate their average radius. B, lysine N-acetylation increases with proximity to a cysteine thiol. Only the closest SG atom to each N-acetylated NZ atom was considered and CysLys pairs were grouped by NZ and SG distance. C, lysine N-acetylation negatively correlates with NZ to SG distance. Data are individual SG to NZ distances for pairs of cysteine and N-acetylated lysine residues <15 Å apart. D, lysine N-acetylation does not correlate with distance to a serine residue. Data are individual NZ to OG distances for pairs of serine and N-acetylated lysine residues <15 Å apart. E, lysine N-acetylation does not correlate with distance to a glutamate residue. Data are individual NZ to closest OE1/OE2 distances for pairs of glutamate and N-acetylated lysine residues <15 Å apart. F, lysine N-acetylation does not correlate with distance to an aspartate residue. Data are individual NZ to closest OD1/OD2 distances for pairs of aspartate and N-acetylated lysine residues <15 Å apart. G, lysine N-acetylation does not correlate with distance to a histidine residue. Data are individual NZ to closest ND1/NE2 distances for pairs of histidine and N-acetylated lysine residues <15 Å apart. H, lysine N-acetylation does not correlate with distance to an arginine residue. Data are individual NZ to closest NH1/NH2 distances for pairs of arginine and N-acetylated lysine residues <15 Å apart. I, lysine N-acetylation does not correlate with distance to another lysine residue. Data are individual NZ to NZ distances for pairs of lysine and N-acetylated lysine residues <15 Å apart. Solid black lines are linear regression lines of the points on each graph with 95% confidence intervals. The red dotted line is the cysteine regression line from Figure S1C.

A

Uniprot	Protein Name	Lys	Cys	Acet	Dist (Å)
Q91VA0	Acyl-coenzyme A synthetase (ACSM1)	200	196	4.73%	8.96
Q8CHR6	Dihydropyrimidine dehydrogenase [$NADP^+$]	384	49	2.89%	5.11
Q8CHR6	Dihydropyrimidine dehydrogenase [$NADP^+$]	384	52	2.89%	5.78
Q9DCM0	Persulfide dioxygenase (ETHE1)	172	170	2.34%	8.61
Q9DCM0	Persulfide dioxygenase (ETHE1)	172	219	2.34%	4.59
O08997	Copper transport protein (ATOX1)	60	12	2.23%	9.22
O08997	Copper transport protein (ATOX1)	60	15	2.23%	4.18

B

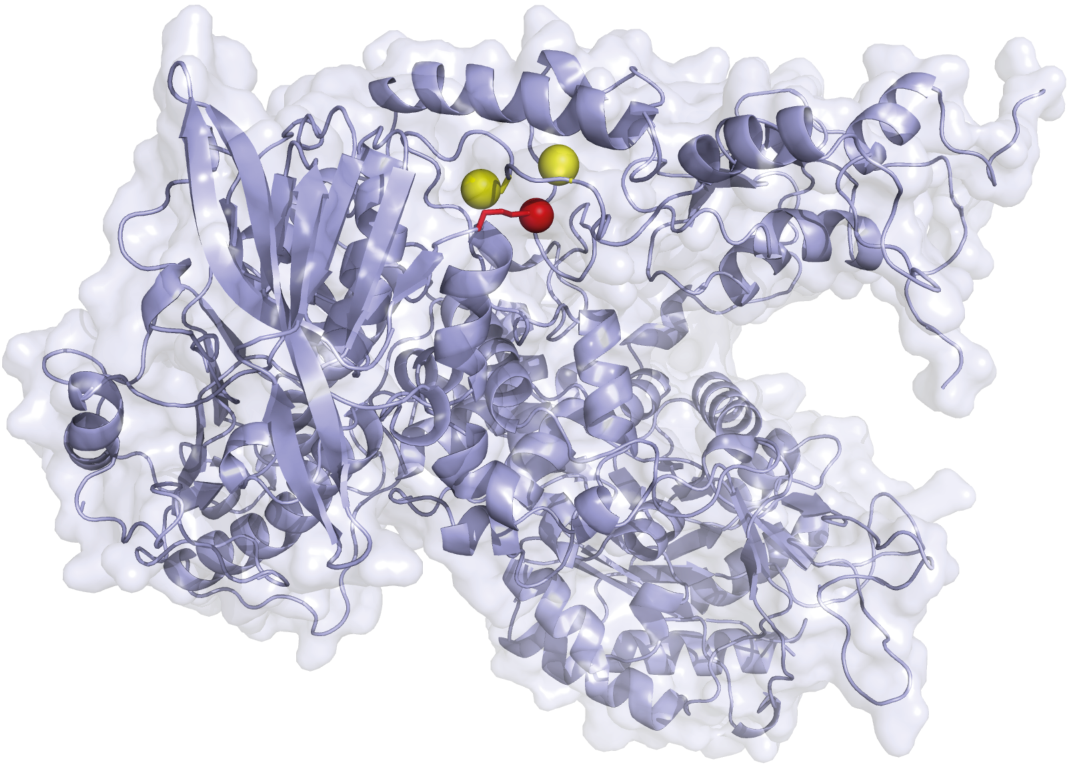

Figure S2. The most N-acetylated CysLys pairs frequently have another nearby cysteine. Related to Figure 2. A, the most N-acetylated lysine residues with a proximal cysteine residue often have additional close cysteine residues. B, a mouse model of pig dihydropyrimidine dehydrogenase (1GTE) contains a lysine residue (red) that can be N-acetylated with two proximal cysteine thiols (yellow).

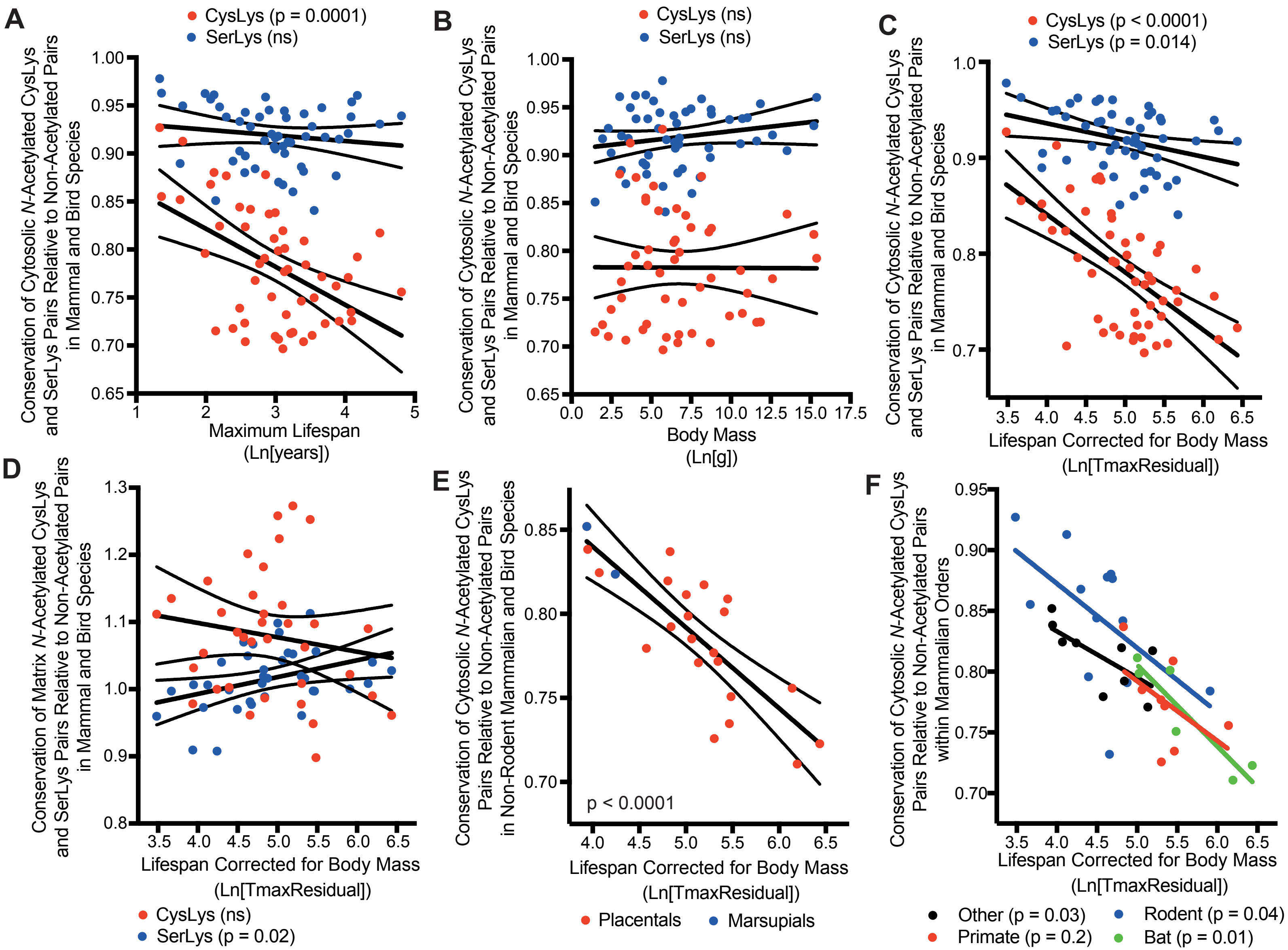

Figure S3. Phylogeny does not explain why conservation N-acetylated CysLys pairs in the cytosol negatively correlates with lifespan. Related to Figure 5. A, conservation of cytosolic N-acetylated CysLys pairs negatively correlates with maximum lifespan in mammals and birds. Data is the conservation of N-acetylated CysLys (red) and SerLys (blue) pairs relative to non-acetylated pairs in 52 mammal and bird species. B, conservation of cytosolic N-acetylated CysLys pairs does not correlate with body mass in mammals and birds. Data is the conservation of N-acetylated CysLys (red) and SerLys (blue) pairs relative to non-acetylated pairs in 52 mammal and bird species. C, conservation of cytosolic N-acetylated CysLys pairs negatively correlates with lifespan corrected for body mass (TmaxResidual) in mammals and birds. TmaxResidual is the maximum lifespan for a species as a percentage of the maximum lifespan expected for a mammal of its body mass. Data is the conservation of N-acetylated CysLys (red) and SerLys (blue) pairs relative to non-acetylated pairs in 52 mammal and bird species. D, conservation of N-acetylated CysLys pairs in the mitochondrial matrix does not correlate with TmaxResidual. Data is the conservation of N-acetylated CysLys (red) and SerLys (blue) pairs relative to non-acetylated pairs in 36 mammal species. E, lower cytosolic N-acetylated CysLys pair conservation is not a consequence of evolutionary distance from mouse. Data is the conservation of cytosolic N-acetylated CysLys pairs relative to non-acetylated CysLys pairs in non-rodent placental (red) and marsupial (blue) species that diverged from mouse >~80 million years ago. Rodent species were excluded to ensure their smaller evolutionary distance from mouse did not cause the correlation with TmaxResidual. The genetically distant marsupial species share low selective pressure against cytosolic acetylated CysLys pairs and short lifespans with mouse. For comparison, mouse ln(TmaxResidual) is 3.94 and relative cytosolic acetylated CysLys pair conservation is 1. F, correlation between cytosolic N-acetylated CysLys pair conservation and TmaxResidual in three mammalian orders. Data is the conservation of cytosolic N-acetylated CysLys pairs in rodents (blue), bats (green) and primates (red) relative to non-acetylated pairs. Other mammals that are not rodents, bats or primates are also shown (black). Lines of best fit are their respective linear regression lines and 95% confidence intervals.

A

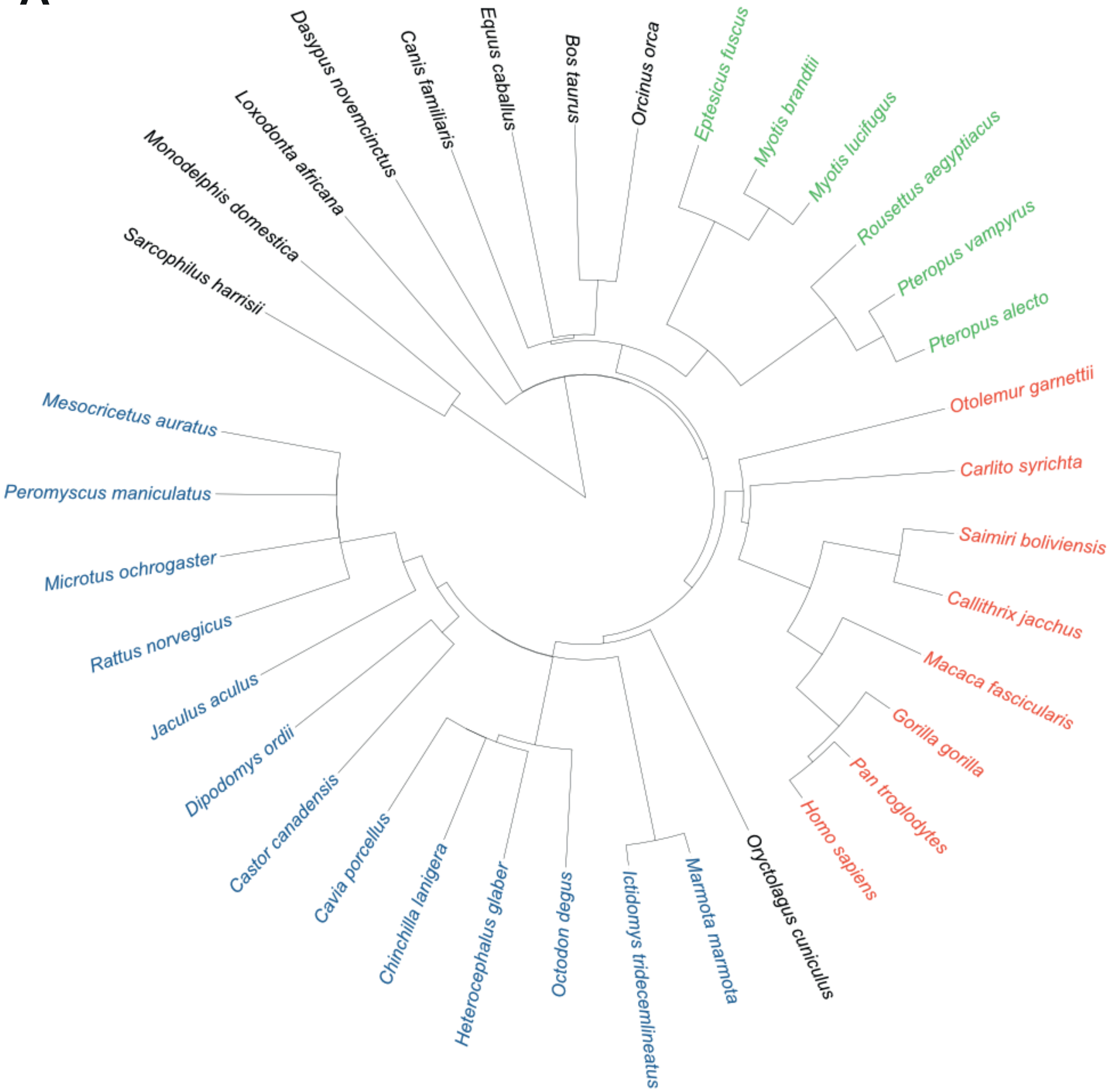

B

	Regression				Phylogenetic Generalized Least Squares (PGLS)		
**Figure 5A**	**y-int**	**slope**	**p-value**	**Pagel's λ**	**y-int**	**slope**	**p-value**
**CysLys**	1.09	-0.058	1.36E-07	0.79	1.00	-0.040	9.00E-04
**SerLys**	0.98	-0.008	0.092	1.02	0.96	-0.006	0.14
**Figure 5C**	**y-int**	**slope**	**p-value**	**Pagel's λ**	**y-int**	**slope**	**p-value**
**Acetylated**	1.22	-0.064	1.18E-06	0.76	1.14	-0.043	0.0019
**Non-acetylated**	1.09	-0.008	0.16	1.05	1.09	0.001	0.72
**Figure 5D**	**y-int**	**slope**	**p-value**	**Pagel's λ**	**y-int**	**slope**	**p-value**
**CysLys - Cys**	1.07	-0.040	2.24E-04	0.81	0.99	-0.027	0.027
**CysLys - Lys**	1.08	-0.023	5.07E-04	0.15	1.02	-0.021	0.0021
**SerLys - Ser**	0.97	-0.003	0.95	0.96	0.96	0.002	0.66
**SerLys - Lys**	0.99	-0.004	0.46	0.82	0.97	-0.003	0.66

Figure S4. Phylogenetic generalised least squares. Related to Figure 5. A, the phylogenetic tree for the phylogenetic generalised least squares (PGLS) method was based on data from the mammalian tree described (Bininda-Emonds et al., 2007). B, the significant correlations of lifespan with cytosolic N-acetylated CysLys pair conservation remain after phylogenetic correction with PGLS. Pagel's λ estimates phylogenetic signal (Pagel, 1999).

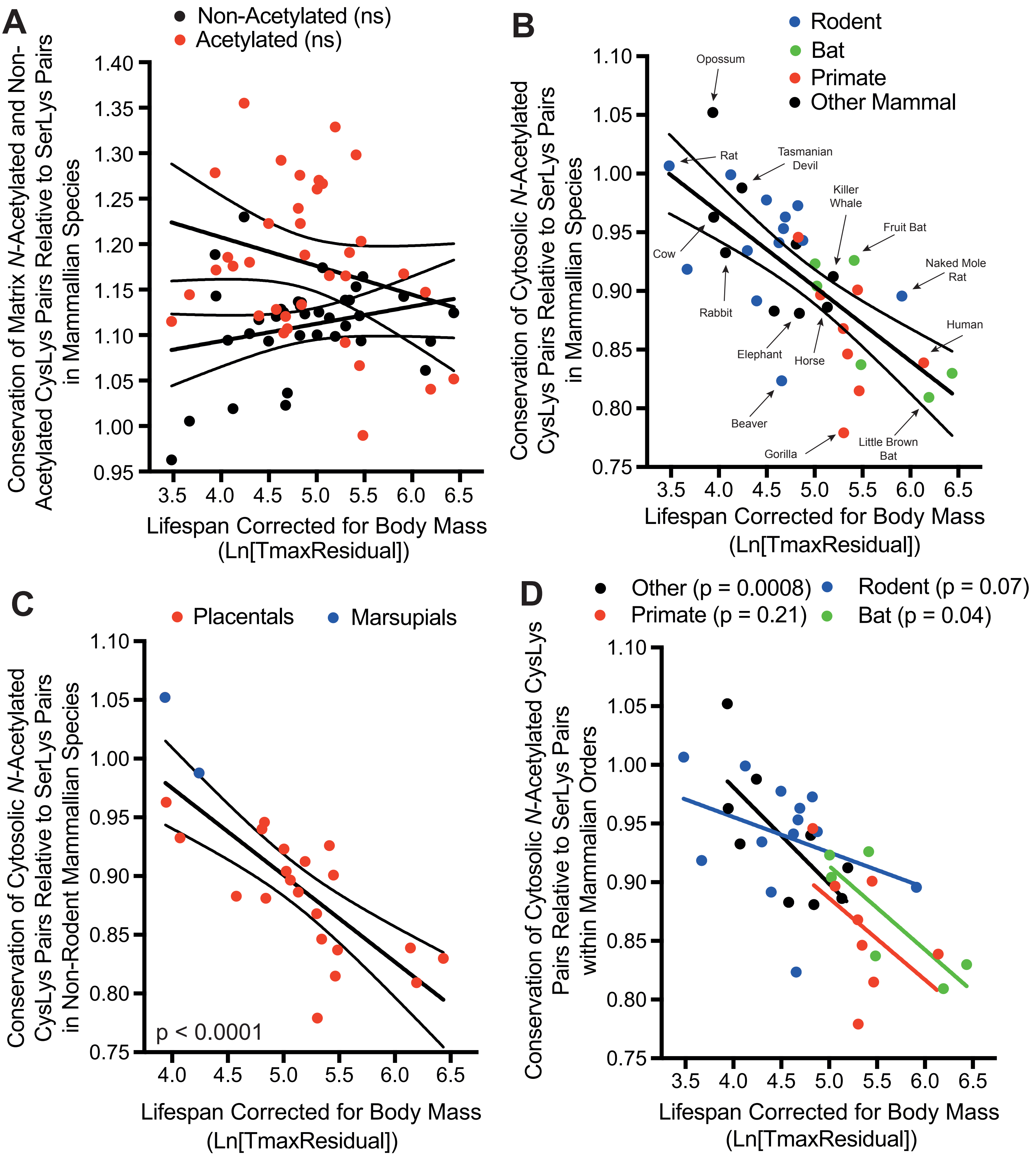

Figure S5. The correlation of lifespan with cytosolic N-acetylated CysLys pair conservation remains when expressed relative to N-acetylated SerLys pairs. Related to Figure 5. TmaxResidual is the maximum lifespan for a species as a percentage of the maximum lifespan expected for a mammal of its body mass. A, conservation of N-acetylated CysLys pairs in the mitochondrial matrix does not correlate with TmaxResidual. Data is the conservation of N-acetylated (red) and non-acetylated (black) CysLys pairs relative to SerLys pairs. B, phylogeny does not explain the correlation between cytosolic N-acetylated CysLys pair conservation and TmaxResidual. Data is N-acetylated CysLys pair conservation in rodents (blue), bats (green), primates (red) and other mammals (black) relative to N-acetylated SerLys pairs. Common names are indicated with scientific names in Table S2. C, lower cytosolic N-acetylated CysLys pair conservation is not a consequence of evolutionary distance from mouse. Data is the conservation of cytosolic N-acetylated CysLys pairs relative to N-acetylated SerLys pairs in non-rodent placental (red) and marsupial (blue) species that diverged from mouse >~80 million years ago. Rodent species were excluded to ensure their smaller evolutionary distance from mouse did not cause the correlation with TmaxResidual. The genetically distant marsupial species share low selective pressure against cytosolic acetylated CysLys pairs and short lifespans with mouse. For comparison, mouse ln(TmaxResidual) is 3.94 and relative cytosolic acetylated CysLys pair conservation is 1. D, correlation between cytosolic N-acetylated CysLys pair conservation and TmaxResidual in three mammalian orders. Data is the conservation of cytosolic N-acetylated CysLys pairs in rodents (blue), bats (green) and primates (red) relative to N-acetylated SerLys pairs. Other mammals that are not rodents, bats or primates are also shown (black). Lines of best fit are their respective linear regression lines and 95% confidence intervals.

SUPPLEMENTAL EXPERIMENTAL PROCEDURES

Creation of mouse structural models – A list of acetylated proteins from mouse liver tissue was obtained from the literature (Weinert et al., 2015). The corresponding protein sequences were taken from UniProt and individually aligned with those in a non-redundant PDB sequence database clustered at 95%, using MODELLER (Webb and Sali, 2016). The best match that had a minimum sequence identity of 50% across their entire length of the query sequence was then used as a structural template. The query sequence was then structurally aligned with the template and this was used to create five predicted structures, with the one with the lowest DOPE score retained for analysis. For each structure the solvent accessible area in $Å^2$ of every atom was calculated using areaimol from the CCP4 software suite (Winn et al., 2011). Distances between atoms of interest were calculated using trigonometry from the 3D coordinates in the generated PDB files.

Definition of cytosolic and mitochondrial matrix proteins – Matrix proteins were classified as those with Gene Ontology annotation (using both human and mouse annotation), present in large-scale matrix APEX tagging study (Rhee et al., 2013) or defined in the matrix compartment of a metabolic model of the mitochondrion (Smith et al.). Cytosolic proteins were defined as those annotated as such in the Gene Ontology (mouse or human) or had been experimentally determined to be cytosolic in the Human Protein Atlas (Thul et al., 2017) (evidence level: validated or supported). Proteins that were dual localized were removed.

Identification of orthologues - For each of the modelled mouse proteins, human orthologs were identified manually using orthology information from the MitoMiner database (Smith and Robinson, 2016). Proteins with no clear one to one human ortholog were removed from the analysis to reduce interference from paralogous proteins. Orthologs of each human protein were identified in 70 additional vertebrates using a BLASTp search against a local BLASTp database containing non-identical protein sequences of each species, with an E-value cut-off of $1e^{-10}$. The top protein hit for each species was considered orthologous if a reciprocal BLASTp search against a database of all human proteins with an assigned gene name from NCBI returned a protein equivalent to the original human protein as top hit (E-value cut-off of $1e^{-10}$). Top hits for each of the species were used to perform local BLASTp searches against a database of human proteins. It was called an ortholog if the reciprocal search returned a protein equivalent to the original human protein (E-value cut-off $1e^{-10}$). Proteins with less than 60 orthologues were removed from the analysis, leaving 442 proteins. Species outlying numbers of identified orthologs (less than lower quartile-1.5*interquartile range) were also removed, leaving 66 species plus mouse for further analysis.

Conservation of residues and pairs - For each gene, protein sequences of identified orthologues were aligned using MUSCLE (default settings) (Edgar, 2004). For each lysine, cysteine or serine identified as part of a surface (>5% $Å^2$) pair with ≤11.5 Å distance between them in mouse, the aligned residue was identified in each other species. Within each species, for each CysLys and SerLys pair, the pair was conserved if both residues exactly matched mouse, not conserved if either residue did not match mouse, and not present if at least one position had no aligned residue. Overall species conservation was calculated as number of conserved pairs/number of present pairs. Calculations were also made for matrix, cytosol, acetylated and non-acetylated pairs and combinations of these. Single residue conservation was analysed in the same manner. Non-acetylated pairs were those pairs identified as being close in the modelled structure and that were not observed to be acetylated by MS.

Maximum lifespan analysis - Maximum recorded lifespan and weight of species was retrieved from the AnAge database for most species (Tacutu et al., 2013). T_{max}Residual was calculated as (maximum lifespan/(4.88*(adult weight$^{0.153}$)))*100, as in the AnAge database.

Statistics and data processing – Statistical analysis was performed in Prism v6. Statistical significance was determined using a two-tailed Student's t-test or one-way ANOVA followed by a Dunnett's multiple comparison test. For linear regression, lines are displayed with their 95% confidence intervals. P-values for linear regression are the probability that the slope of the regression line is zero and there is no correlation. Differences in frequency were tested using two-sided Chi-square tests.

Phylogenetic generalised least squares - The phylogenetic tree was based on data from the mammalian tree described (Bininda-Emonds et al., 2007). As *Sarcophilus harrisii* was not present in the tree, the position of the closely related *Sarcophilus laniarius* from the same genus was used to represent this species. The phylogenetic generalised least squares method (Grafen, 1989) was implemented using the R package nlme to correct for phylogenetic bias, using Pagel's λ (Pagel, 1999) to estimate phylogenetic signal.

REFERENCES

Bininda-Emonds, O.R., Cardillo, M., Jones, K.E., MacPhee, R.D., Beck, R.M., Grenyer, R., Price, S.A., Vos, R.A., Gittleman, J.L. & Purvis, A. (2007). The delayed rise of present-day mammals. Nature *446*, 507-12.

Edgar, R.C. (2004). Muscle: Multiple sequence alignment with high accuracy and high throughput. Nucleic Acids Res *32*, 1792-7.

Grafen, A. (1989). The phylogenetic regression. Philos Trans R Soc Lond B Biol Sci *326*, 119-57.

Pagel, M. (1999). Inferring the historical patterns of biological evolution. Nature *401*, 877-84.

Rhee, H.W., Zou, P., Udeshi, N.D., Martell, J.D., Mootha, V.K., Carr, S.A. & Ting, A.Y. (2013). Proteomic mapping of mitochondria in living cells via spatially restricted enzymatic tagging. Science *339*, 1328-1331.

Smith, A.C., Eyassu, F., Mazat, J.-P. & Robinson, A.J. Mitocore: A curated constraint-based model for simulating human central metabolism. BMC Systems Biology *In Press*.

Smith, A.C. & Robinson, A.J. (2016). Mitominer v3.1, an update on the mitochondrial proteomics database. Nucleic Acids Res *44*, D1258-61.

Tacutu, R., Craig, T., Budovsky, A., Wuttke, D., Lehmann, G., Taranukha, D., Costa, J., Fraifeld, V.E. & de Magalhaes, J.P. (2013). Human ageing genomic resources: Integrated databases and tools for the biology and genetics of ageing. Nucleic Acids Res *41*, D1027-33.

Thul, P.J., Akesson, L., Wiking, M., Mahdessian, D., Geladaki, A., Ait Blal, H., Alm, T., Asplund, A., Bjork, L., Breckels, L.M., et al. (2017). A subcellular map of the human proteome. Science *356*.

Webb, B. & Sali, A. (2016). Comparative protein structure modeling using modeller. Curr Protoc Protein Sci *86*, 2.9.1-2.9.37.

Weinert, B.T., Moustafa, T., Iesmantavicius, V., Zechner, R. & Choudhary, C. (2015). Analysis of acetylation stoichiometry suggests that sirt3 repairs nonenzymatic acetylation lesions. EMBO J *34*, 2620-32.

Winn, M.D., Ballard, C.C., Cowtan, K.D., Dodson, E.J., Emsley, P., Evans, P.R., Keegan, R.M., Krissinel, E.B., Leslie, A.G., McCoy, A., et al. (2011). Overview of the ccp4 suite and current developments. Acta Crystallogr D Biol Crystallogr *67*, 235-42.
