# Supplementary material for: Proximal Cysteines that Enhance Lysine N-Acetylation of Cytosolic Proteins in Mice Are Less Conserved in Longer-Living Species
Source: Cell Rep. 2018 Aug 7;24(6):1445–55. doi: 10.1016/j.celrep.2018.07.007 (PMC6092265; doi:10.1016/j.celrep.2018.07.007)
Supplement: Document S2. Article plus Supplemental Information [file mmc7.pdf]

Cell Reports

Report

Proximal Cysteines that Enhance Lysine *N*-Acetylation of Cytosolic Proteins in Mice Are Less Conserved in Longer-Living Species

Graphical Abstract

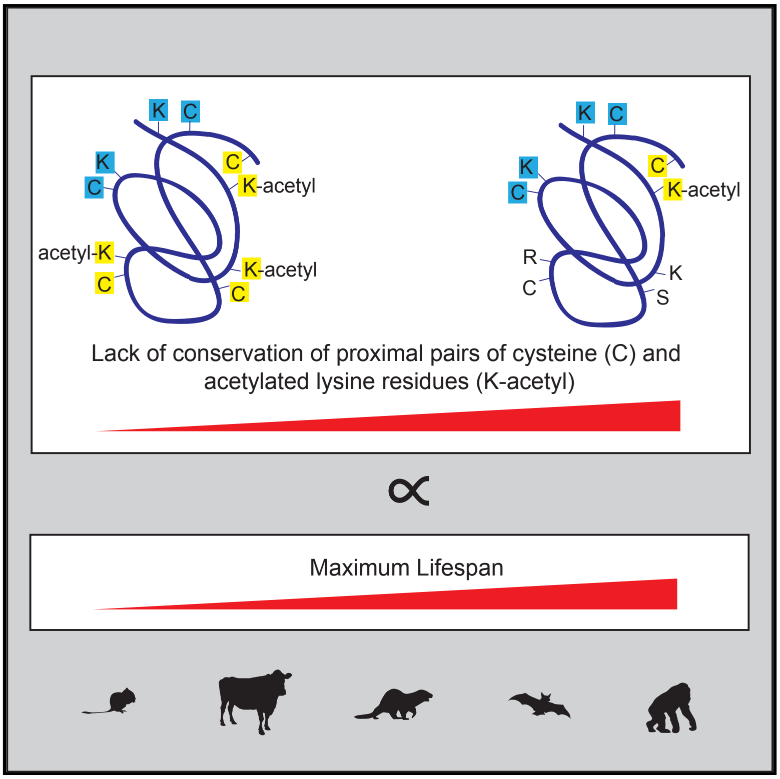

Authors

Andrew M. James, Anthony C. Smith, Cassandra L. Smith, Alan J. Robinson, Michael P. Murphy

Correspondence

aj@mrc-mbu.cam.ac.uk (A.M.J.), mpm@mrc-mbu.cam.ac.uk (M.P.M.)

In Brief

Acetyl-CoA non-enzymatically *N*-acetylates protein lysines. Using proteins *N*-acetylated in mouse liver, James et al. show *N*-acetylation is greater if a cysteine is within ~10 Å. These pairs of proximal cysteine and *N*-acetylated lysines are less conserved in species with longer lifespans. This might explain how dietary restriction extends lifespan.

Highlights

- Creation of a mouse structural library of *in vivo* lysine *N*-acetylation on 619 proteins
- Proximal cysteines enhance protein lysine *N*-acetylation *in vivo*
- Proximal *N*-acetylated lysine and cysteine residues are less conserved
- Conservation of these proximal residues is lower in species with longer lifespans

James et al., 2018, Cell Reports *24*, 1445–1455
August 7, 2018 © 2018 The Author(s).
https://doi.org/10.1016/j.celrep.2018.07.007

CellPress

OPEN ACCESS
CellPress

Proximal Cysteines that Enhance Lysine *N*-Acetylation of Cytosolic Proteins in Mice Are Less Conserved in Longer-Living Species

Andrew M. James,[1,*] Anthony C. Smith,[1,2] Cassandra L. Smith,[1,2] Alan J. Robinson,[1] and Michael P. Murphy[1,3,*]
[1]Medical Research Council Mitochondrial Biology Unit, University of Cambridge, Cambridge CB2 0XY, UK
[2]These authors contributed equally
[3]Lead Contact
*Correspondence: aj@mrc-mbu.cam.ac.uk (A.M.J.), mpm@mrc-mbu.cam.ac.uk (M.P.M.)
https://doi.org/10.1016/j.celrep.2018.07.007

SUMMARY

Acetyl-coenzyme A (CoA) is an abundant metabolite that can also alter protein function through non-enzymatic *N*-acetylation of protein lysines. This *N*-acetylation is greatly enhanced *in vitro* if an adjacent cysteine undergoes initial *S*-acetylation, as this can lead to *S*→*N* transfer of the acetyl moiety. Here, using modeled mouse structures of 619 proteins *N*-acetylated in mouse liver, we show lysine *N*-acetylation is greater *in vivo* if a cysteine is within ∼10 Å. Extension to the genomes of 52 other mammalian and bird species shows pairs of proximal cysteine and *N*-acetylated lysines are less conserved, implying most *N*-acetylation is detrimental. Supporting this, there is less conservation of cytosolic pairs of proximal cysteine and *N*-acetylated lysines in species with longer lifespans. As acetyl-CoA levels are linked to nutrient supply, these findings suggest how dietary restriction could extend lifespan and how pathologies resulting from dietary excess may occur.

INTRODUCTION

Acetyl-coenzyme A (CoA) and other acyl-CoAs are central metabolites in the oxidation of carbohydrate and fat in the mitochondrial matrix, as well as providing the building blocks for fatty acid synthesis in the cytosol (Pietrocola et al., 2015). However, these metabolites also impact cellular function by *N*-acylating the ε-amino of protein lysines. The significance of *N*-acylation is implied by the existence of several sirtuins (Sirt1–7), which use NAD^+ to remove acetyl and other acyl groups from protein lysines and are important in the pathology of a wide range of degenerative diseases, including cancer, aging, and diabetes (McDonnell et al., 2015; Pan and Finkel, 2017). *N*-acetylation was originally considered to be solely a regulatory modification, allowing the cell to respond to acetyl-CoA, the acetyl-CoA/CoA ratio, or NAD^+. This was reassessed after observation of several thousand sites of lysine *N*-acetylation *in vivo* (Rardin et al., 2013; Weinert et al., 2015; Baeza et al., 2016), with the vast majority having a very low (∼0.1%) stoichiometry of acetylation (Weinert et al., 2015, 2017; James et al., 2017). Lysines of mitochondrial proteins can be non-enzymatically *N*-acetylated *in vitro* by acetyl-CoA (Wagner and Payne, 2013; James et al., 2017), and other acyl-CoAs, such as succinyl-CoA, malonyl-CoA, and glutaryl-CoA, also generate *N*-linked modifications on lysines *in vivo* without known transferase enzymes (Weinert et al., 2013; Peng et al., 2011; Tan et al., 2014). Consequently, it was proposed that acyl-CoAs represent a "carbon stress," through which chronic exposure to acyl-CoAs causes cumulative cellular damage that contributes to degenerative diseases and aging, as well as explaining the benefits of sirtuins and dietary restriction (Wagner and Hirschey, 2014; Trub and Hirschey, 2018; Weinert et al., 2017).

Non-enzymatic *N*-acylation occurs when the amine group (pK_a ∼10.5) of a protein lysine deprotonates to become a nucleophile, which then attacks the thioester carbonyl of an acyl-CoA to generate a stable amide-linked modification (Wagner and Payne, 2013). However, lysines are not the only nucleophilic residues that can react with acyl-CoAs (Bizzozero et al., 2001; James et al., 2017). The deprotonated thiol of a cysteine (pK_a ∼8.5) can be non-enzymatically *S*-acylated, and *in vitro* cysteine *S*-acetylation by acetyl-CoA is ∼100-fold more rapid than the corresponding *N*-acetylation of a lysine (James et al., 2017). However, although cysteine reactivity is greater, the thioester bond of an *S*-acyl cysteine is less stable and prone to further nucleophilic attack. In this way, *S*-acylated cysteines can cause *N*-acylation of nearby lysines via subsequent *S*→*N* transfer of their acyl moiety (James et al., 2017; Cohen et al., 2013). The progression of this *S*→*S*→*N*-acyl transfer reaction has been shown *in vitro* on a synthetic peptide and on mitochondrial membrane proteins (James et al., 2017). Furthermore, protein *N*-acetylation is diminished by cysteine alkylation (James et al., 2017), cysteine mutation prevents auto-catalytic lysine *N*-acetylation of Tau protein (Cohen et al., 2013), and sites of lysine *N*-acetylation and cysteine *S*-acylation are often observed on the same peptide in mouse liver (Rardin et al., 2013; Gould et al., 2015; James et al., 2017). There are also rapid enzymatic and non-enzymatic examples of similar *S*→*S*→*N* transfer reactions, where the second rate-limiting step is enhanced by proximity, including native chemical ligation and ubiquitin ligation.

Although examples of this chemistry exist, the extent that surface cysteines generally enhance lysine *N*-acetylation *in vivo*, in

This is an open access article under the CC BY license (http://creativecommons.org/licenses/by/4.0/).

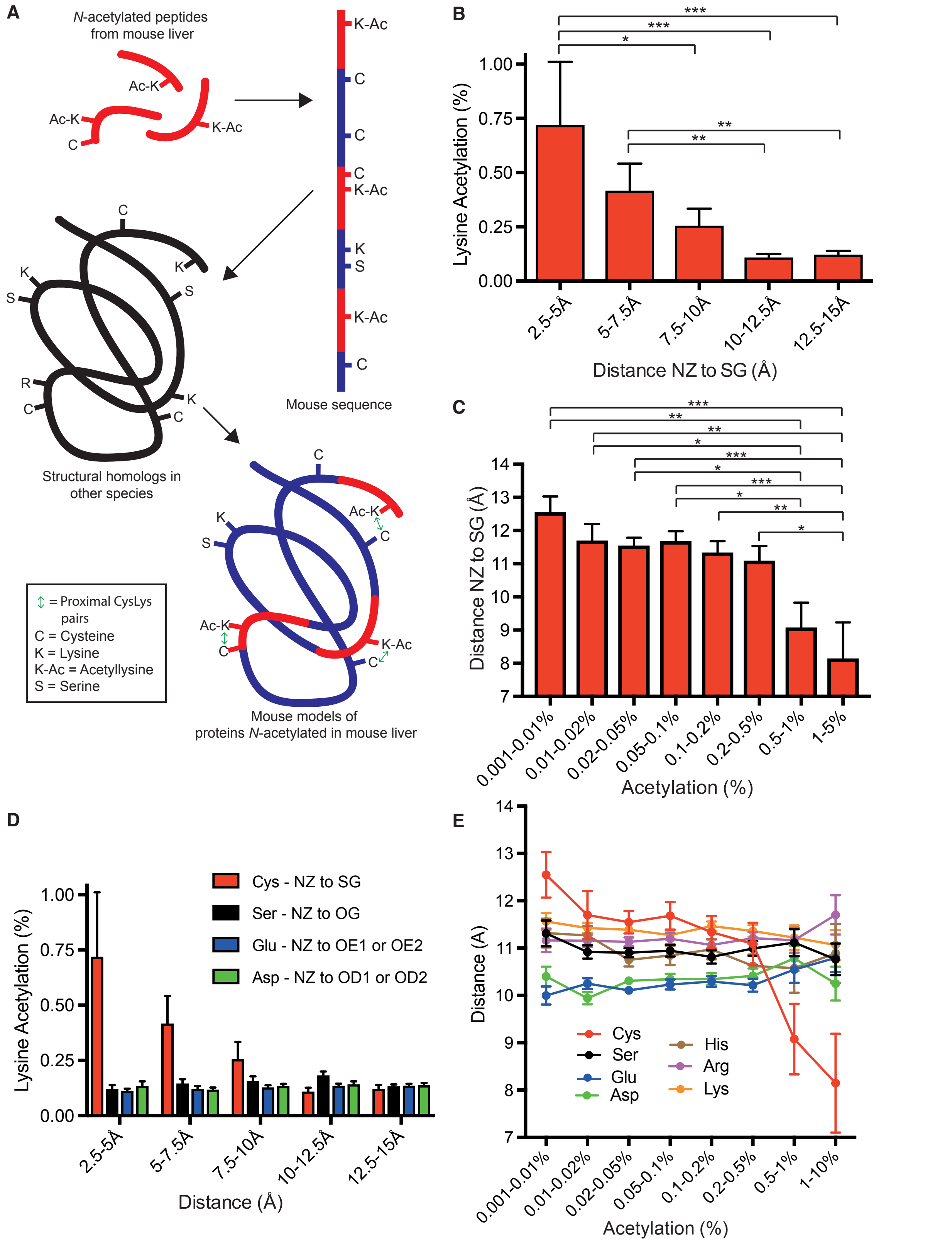

(legend on next page)

the presence of pathways that might mitigate against it, is unclear. Here, we explore whether surface cysteines enhance lysine *N*-acetylation *in vivo*, using an existing mouse liver proteomic dataset that reports the stoichiometry of lysine *N*-acetylation at each site (Weinert et al., 2015). From these *N*-acetylated peptides and the crystal structures of homologous proteins, we generated mouse structural models of 619 proteins that are *N*-acetylated *in vivo* and show that lysine *N*-acetylation is increased by proximity to a surface cysteine. Furthermore, we found decreased conservation of pairs of proximal cysteine and *N*-acetylated lysines (CysLys) in the genomes of 52 other mammal and bird species. The low conservation of *N*-acetylated CysLys pairs strongly correlated with maximal lifespan, and this was not observed for proximal pairs of serine and *N*-acetylated lysines (SerLys). Our results support a model where lysine *N*-acetylation is a non-enzymatic byproduct of high concentrations of acetyl-CoA, with effects that favor decreased conservation of *N*-acetylated CysLys pairs in long-lived species.

RESULTS

Generating a 3D Structural Library of Mouse Liver Proteins with *N*-Acetylated Lysines

Primary protein sequences are often searched for motifs that increase reactivity of certain residues *in vivo*, but the proximity of two residues cannot consistently be predicted like this. As molecular structures for many proteins now exist, we created a dataset of distances between *N*-acetylated lysines and nearby surface cysteines to see whether cysteines influence lysine *N*-acetylation *in vivo* (Figure 1A).

We began with a stoichiometric dataset that identified 4,320 *N*-acetylated lysines in mouse liver (Weinert et al., 2015; Baeza et al., 2016). We took the protein sequences of the *N*-acetylated peptides from this and built single-subunit models of the mouse proteins using the most homologous existing structure as a template (Fiser and Sali, 2003). Modeling was required as *N*-acetylated lysines, and their proximal cysteines were often absent from homologous structures in other species. For each model, we calculated distances from the amine nitrogen atom (NZ) of all lysines to the thiol sulfur atom (SG) of all cysteines. This created 147,880 NZ to SG distances on 619 proteins that could be correlated with the initial stoichiometric acetylation dataset (Weinert et al., 2015). As only surface residues can be *N*-acetylated, we considered only pairs of cysteine and lysine (CysLys) residues where both the NZ or SG atoms were solvent accessible (>5 $Å^2$) in the modeled single subunit. This 5 $Å^2$ cutoff is ~7% of their potential solvent accessible surface area, as the maximum observed for NZ and SG atoms was 64.7 $Å^2$ and 74.5 $Å^2$, respectively (Table S1). Although 88.8% of lysine amines were exposed, 61.2% of cysteine thiols were buried internally. This left 52,321 CysLys pairs in which both the NZ and SG atoms were on the surface of the modeled subunit.

The 619 proteins have an average solvent-accessible surface area of 20,862 $Å^2$, corresponding to a sphere with a radius of ~40 Å. At this size, interaction between two surface residues more than ~15 Å apart is often blocked by intervening surface features or by curvature of the protein surface (Figure S1A). Thus, analysis was limited to 2,595 CysLys pairs where NZ and SG atoms were <15 Å apart (Table S1). Of these pairs, 376 were *N*-acetylated on their lysine to some degree (average acetylation = 0.19%). Supporting our structural approach for finding motifs, only 124 of the 376 (33%) acetylated CysLys pairs that were <15 Å apart also lay within 10 residues on the primary sequence (median = 27 residues separation; Table S1).

Of the 376 *N*-acetylated CysLys pairs, 48 of the cysteines (12.7%) had previously been identified as *S*-acylated (Gould et al., 2015). This is significantly higher than non-*N*-acetylated pairs, where only 118 of the remaining 2,219 (5.3%) have an *S*-acylated cysteine ($p < 0.0001$; Table S1). This is consistent with *S*→*S*-acyl exchange from acetyl-CoA to a protein cysteine followed by *S*→*N*-acyl transfer to a nearby lysine.

Close Proximity of a Cysteine Enhances Lysine *N*-Acetylation *In Vivo*

One prediction of our model is that the closer cysteines and lysines are, the greater the extent of lysine acetylation. When the 376 CysLys pairs <15 Å apart (Table S1) were grouped by their NZ to SG distances, there was a significant increase in lysine *N*-acetylation when a cysteine was <7.5 Å away, and this declined with distance (Figure 1B). As each surface lysine is paired with every surface cysteine on the protein, the prior analysis filtered out distant CysLys pairs (>15 Å). To show Figure 1B was not an artifact of filtering, a further dataset was created with only the distance from each lysine to the nearest surface cysteine (Table S1; Figure S1B). When these 2,387 CysLys pairs were grouped by NZ to SG distances, there was again a significant increase in lysine *N*-acetylation when CysLys pairs were <7.5 Å apart. Finally, to confirm the result was independent of how the dataset was grouped, the initial 376 CysLys pairs were grouped by their degree of *N*-acetylation (Table S1). The two most *N*-acetylated groups (>0.5%) were significantly closer than the ~11.5 Å separation observed with less acetylated CysLys pairs (Figure 1C). Plotting individual CysLys pairs also showed a significant correlation between lysine *N*-acetylation

Figure 1. Protein Lysine *N*-Acetylation Is Increased by Proximity to a Cysteine

(A) Creation of structural library of proteins with *N*-acetylated lysines in mouse liver *in vivo*. The set of 4,320 *N*-acetylated peptides is from Weinert et al. (2015). Mouse structural models of 619 proteins with *N*-acetylated lysines were generated based on molecular structures in other species, and the distance between their lysine amine (NZ) and cysteine thiol (SG) atoms was calculated.

(B) Lysine *N*-acetylation increases with proximity to a cysteine thiol. All *N*-acetylated CysLys pairs <15 Å apart were grouped by NZ to SG distance.

(C) The most *N*-acetylated lysines are closer to cysteine thiols. All *N*-acetylated CysLys pairs <15 Å apart were grouped by their degree of *N*-acetylation.

(D) Lysine *N*-acetylation is not caused by serines, glutamates, or aspartates. Pairs <15 Å apart were grouped by their NZ to SG, OG, OE1/OE2, or OD1/OD2 distances.

(E) The most *N*-acetylated lysines are not closer to serines, glutamates, aspartates, histidines, arginines, or other lysines. Pairs <15 Å apart were grouped by their degree of *N*-acetylation.

Data are the mean ± SEM. $^{*}p < 0.05$; $^{**}p < 0.01$; $^{***}p < 0.001$; ns, not significant.

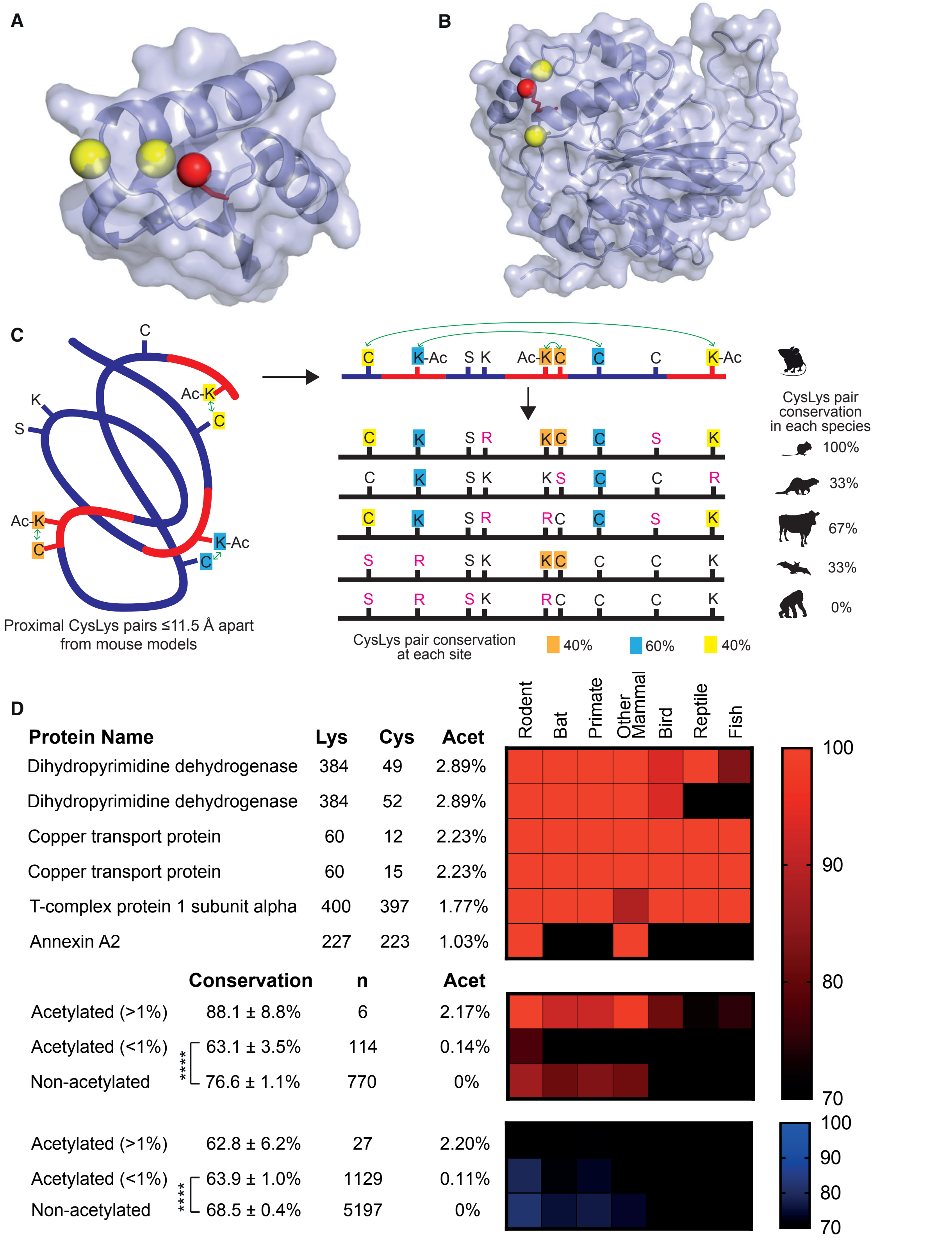

(legend on next page)

and NZ to SG distance (Figure S1C). In summary, cysteines enhance lysine *N*-acetylation *in vivo*, and this decays to background when separation is >10–11.5 Å (Figures 1B, 1C, S1B, and S1C).

Proximity to Other Charged Residues Does Not Enhance Lysine *N*-Acetylation *In Vivo*

To show it is the reactive thiol of a cysteine that promotes lysine *N*-acetylation, we assessed the effect of serines on lysine *N*-acetylation. Serines are structurally identical to cysteines, except the reactive sulfur (SG) atom of cysteine is an oxygen atom (OG) that will not lead to lysine *N*-acetylation. As expected, the NZ to OG distance did not correlate with lysine *N*-acetylation, consistent with thiol reactivity leading to lysine *N*-acetylation (Table S1; Figures 1D, 1E, and S1D). In the active sites of enzymes, charged amino acids can enhance the reactivity of nucleophiles by stabilizing their deprotonated form. However, proximity of a lysine to the acidic moieties of glutamate or aspartate, or to the basic groups of histidine, arginine, and lysine, showed no correlation with lysine *N*-acetylation (Table S1; Figures 1D, 1E, and S1E–S1I).

Most Proximal *N*-Acetylated CysLys Pairs Are Less Conserved

Three of the four most *N*-acetylated CysLys pairs <11.5 Å apart in the CysLys dataset have a second proximal cysteine <11.5 Å away (Table S1; Figures 2A, 2B, S2A, and S2B). Although the presence of multiple cysteines in close proximity could suggest *N*-acetylation of these lysines is functional, their low stoichiometry of *N*-acetylation *in vivo* argues otherwise. This illustrates an unresolved dichotomy in the field: the extent to which lysine *N*-acetylation is a regulatory modification or an unintentional byproduct of high *in vivo* concentrations of acetyl-CoA (Wagner and Hirschey, 2014; Trub and Hirschey, 2018).

One way to investigate this dichotomy, while incorporating the low *N*-acetylation stoichiometry present *in vivo* across a large range of acetylation sites, is to evaluate the conservation of CysLys pairs in diverse genomes (Figure 2C). CysLys pairs where regulatory modifications occur should be conserved, and sites of non-functional or detrimental acylation should be neutral or comparatively less conserved. For this, *N*-acetylated mouse proteins were aligned to their orthologs in 66 further vertebrate species (13 rodents, 6 bats, 8 primates, 9 other mammalian species, 16 birds, 7 reptiles, and 7 fish), and each of the 890 CysLys pairs was considered conserved only if both the cysteine and lysine were present in the ortholog (Table S2). Individually, the most acetylated (>1%) proximal CysLys pairs with identifiable orthologs were conserved within vertebrates (Figure 2D). This small group was more conserved across all 66 vertebrate species (88.1% ± 8.8%; n = 6), although not significantly so, than either the 114 weakly acetylated (<1%) proximal CysLys pairs (63.1% ± 3.5%), the 770 proximal CysLys pairs not observed as acetylated by mass spectrometry (MS) (non-acetylated; 76.6% ± 1.1%), or the 27 highly acetylated (>1%) proximal SerLys pairs (62.8% ± 6.2%). Strikingly, the weakly *N*-acetylated (<1%) group that contains 95% of proximal *N*-acetylated CysLys pairs was significantly less conserved than the non-acetylated proximal CysLys pairs (Figure 2D; $p < 0.0001$).

Thus, although selection of proximal cysteines to enhance lysine *N*-acetylation may occur in a few places, for the vast majority of sites, lysine *N*-acetylation at best serves no purpose and at worst negatively impacts the organism.

Increased *N*-Acetylation in Matrix Leads to Compensatory Changes to CysLys Pairs

The higher acetyl-CoA concentration and pH of the mitochondrial matrix make lysines in that compartment more susceptible to acetylation than those in the cytosol (Figure 3A) (Wagner and Payne, 2013; James et al., 2017). This effect was noted in the original study (Weinert et al., 2015), and our re-analysis shows 20.1% (41/204) of proximal CysLys pairs are *N*-acetylated in the matrix compared to 13.4% of proximal CysLys pairs (90/669) in the cytosol ($p = 0.02$). Furthermore, for mitochondrial proteins, the average *N*-acetylation of CysLys pairs and SerLys pairs is 2.6-fold and 5.2-fold higher, respectively, than those in the cytosol (Figure 3B).

If most low-level lysine *N*-acetylation negatively impacts organismal fitness, then adaptation of cytosolic and mitochondrial proteins in response to increased *N*-acetylation may have occurred (Figure 3A). Consistent with this, the surface density of exposed lysine (NZ) and cysteine (SG) atoms was 13.2% and 3.4% lower, respectively, in the mitochondrial matrix relative to the cytosol (Figure 3C; Table S3). These changes were matched by 8.8% and 5.1% increases in the surface density of less reactive arginine (NH1/NH2) and serine (OG) atoms of matrix proteins. These differences were reflected in a 29.5% decrease in the surface density of potentially reactive (<11.5 Å) CysLys pairs in the matrix. Where proximal CysLys pairs exist, they are significantly further apart by 0.42 Å and 0.29 Å than corresponding SerLys pairs in the matrix ($p = 0.015$) and cytosol ($p = 0.001$), respectively (Figure 3D; see also Figure 1E). This may prevent some interactions and, where interaction is possible, this ~3% increase in distance (Å) may lower the *S*-acetyl cysteine concentration (Å^{-3}) near lysines by ~10%. Finally, solvent exposure of the SG atom of proximal CysLys pairs was 11.5% lower in the matrix than in the cytosol ($p = 0.0008$; Figure 3E).

Figure 2. Most Proximal *N*-Acetylated CysLys Pairs Are Not Conserved

(A) A mouse model of human persulfide dioxygenase (4CHL) contains a lysine amine (red) that is *N*-acetylated flanked by two cysteine thiols (yellow).

(B) A mouse model of human copper transport protein (3IWL) contains a lysine amine (red) that is *N*-acetylated near two cysteine thiols (yellow).

(C) Schematic diagram for determining the degree to which proximal (≤11.5 Å) CysLys pairs are conserved. A CysLys pair is conserved only if both residues were present. Conservation of CysLys pairs at specific sites in a range of species is depicted vertically and conservation at a range of sites in a particular species is shown horizontally.

(D) A few *N*-acetylated sites are conserved, but most are not. A heatmap of genomic conservation of individual CysLys (red) pairs *N*-acetylated by >1% in mouse in 66 vertebrate species. These have been grouped and compared to less acetylated CysLys pairs (<1%) and non-acetylated CysLys pairs. A heatmap of genomic conservation of SerLys pairs (blue) is shown. ****$p < 0.0001$.

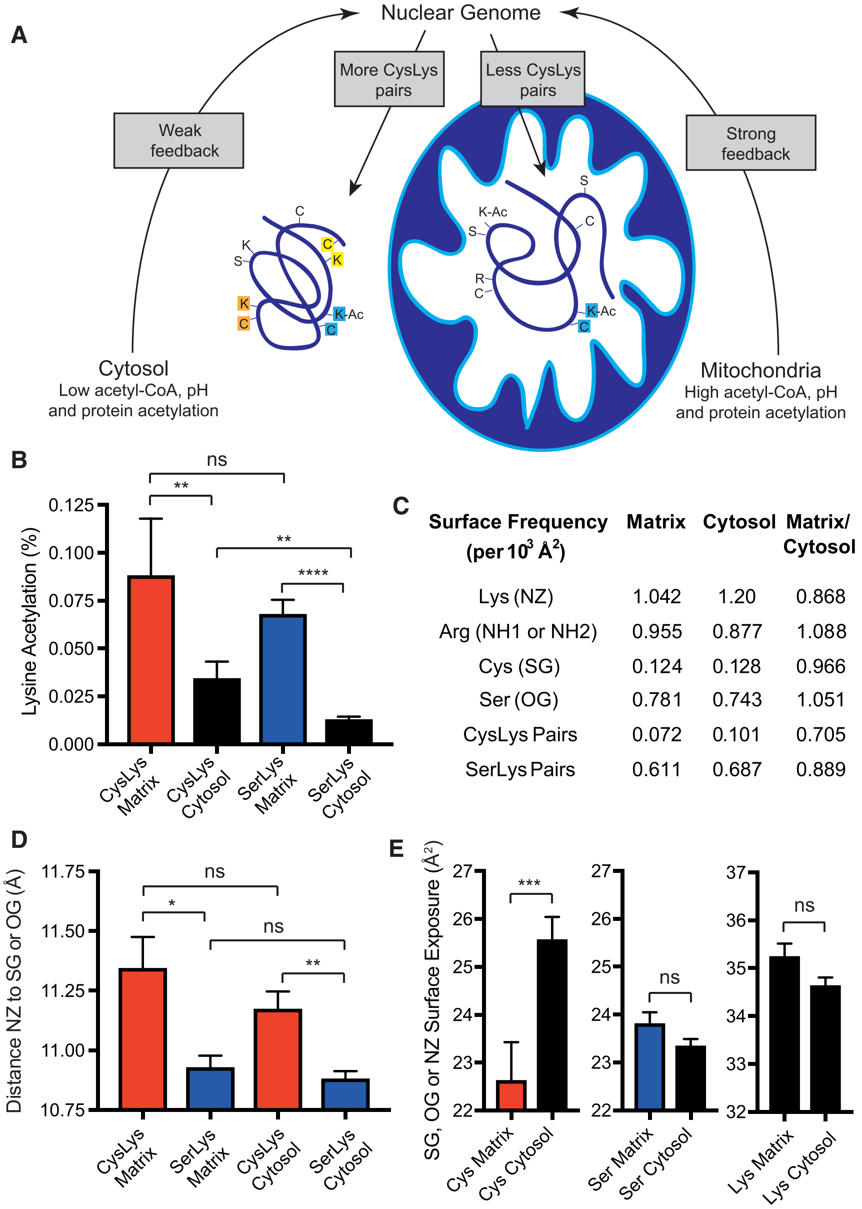

C

Surface Frequency (per 10^3 Å^2)	Matrix	Cytosol	Matrix/ Cytosol
Lys (NZ)	1.042	1.20	0.868
Arg (NH1 or NH2)	0.955	0.877	1.088
Cys (SG)	0.124	0.128	0.966
Ser (OG)	0.781	0.743	1.051
CysLys Pairs	0.072	0.101	0.705
SerLys Pairs	0.611	0.687	0.889

Figure 3. Loss of Proximal CysLys Pairs from the Mitochondrial Matrix

(A) CysLys pairs may be lost from matrix proteins.

(B) The mitochondria are a more acetylating environment than the cytosol. All CysLys and SerLys pairs <15 Å apart were segregated by cellular location.

(C) Surface lysine and cysteines and CysLys pairs are less frequent in the matrix.

(D) When present, CysLys pairs are further apart than SerLys pairs. All CysLys and SerLys pairs <15 Å apart were segregated by cellular location.

(E) The cysteine of CysLys pairs is less solvent exposed in the matrix. SG and OG atom surface exposure is from CysLys and SerLys pairs, and NZ surface exposure is from both CysLys and SerLys pairs.

Data are the mean ± SEM. $^*p < 0.05$; $^{**}p < 0.01$; $^{***}p < 0.001$; $^{****}p < 0.0001$.

This is consistent with increased lysine *N*-acylation in the matrix causing compensatory changes in mitochondrial protein to limit lysine *N*-acylation.

N-Acetylated Cytosolic CysLys Pairs Are Also Less Conserved

The above comparison cannot explore whether proximal cytosolic *N*-acetylated CysLys pairs are also detrimental, so we next considered conservation of CysLys pairs in each species (Figure 4A; see also Figure 2C). This analysis was kept to 36 mammalian (13 rodent, 6 bat, 8 primate, and 9 other mammalian species) and 16 bird species whose body temperature is similar as non-enzymatic acetylation is temperature dependent (James et al., 2017). Within species, CysLys pairs observed as *N*-acetylated in mouse were less conserved than CysLys pairs not observed as acetylated by MS (Figure 4B), and CysLys pairs of matrix proteins were less conserved than CysLys pairs of cytosolic proteins (Figure 4B). When Figure 4B is further partitioned, *N*-acetylation of cytosolic proteins is also detrimental (Figure 4C).

Genetic distance from mouse is a confounding variable, so conservation of proximal *N*-acetylated CysLys pairs (≤11.5 Å) was expressed relative to that of proximal CysLys pairs where the lysine was not observed as *N*-acetylated by MS (Figure 4A). If *N*-acetylated and non-acetylated pairs experience similar selective pressures, they will evolve equally and their relative conservation values will be ∼1. Differences between *N*-acetylated and non-acetylated CysLys and SerLys pairs do not occur for mitochondrial matrix proteins, as their relative conservation values are ∼1 (Figure 4D). In contrast, acetylated CysLys pairs were less conserved than non-acetylated CysLys pairs on cytosolic proteins ($p = 5.5 \times 10^{-29}$; Figure 4D). The basis for this result can be seen in a plot of the individual species, where the conservation rate deviates from 45° with all 52 species lying below this line (Figure 4E). This relative conservation rate for cytosolic CysLys pairs was lower than for SerLys pairs (Figures 4D and 4E). This equates to greater mutation of *N*-acetylated cytosolic CysLys pairs (87.1% ± 4.1%) and SerLys pairs (23.5% ± 1.0%) than their non-acetylated counterparts (Figure 4D). Individual cysteines are often functional, and protein cysteines as a group form a large redox buffer (Requejo et al., 2010). Consistent with this, cytosolic non-acetylated as well as matrix *N*-acetylated and non-acetylated CysLys pairs are all more conserved than corresponding SerLys pairs (Figure 4F). In contrast, conservation of

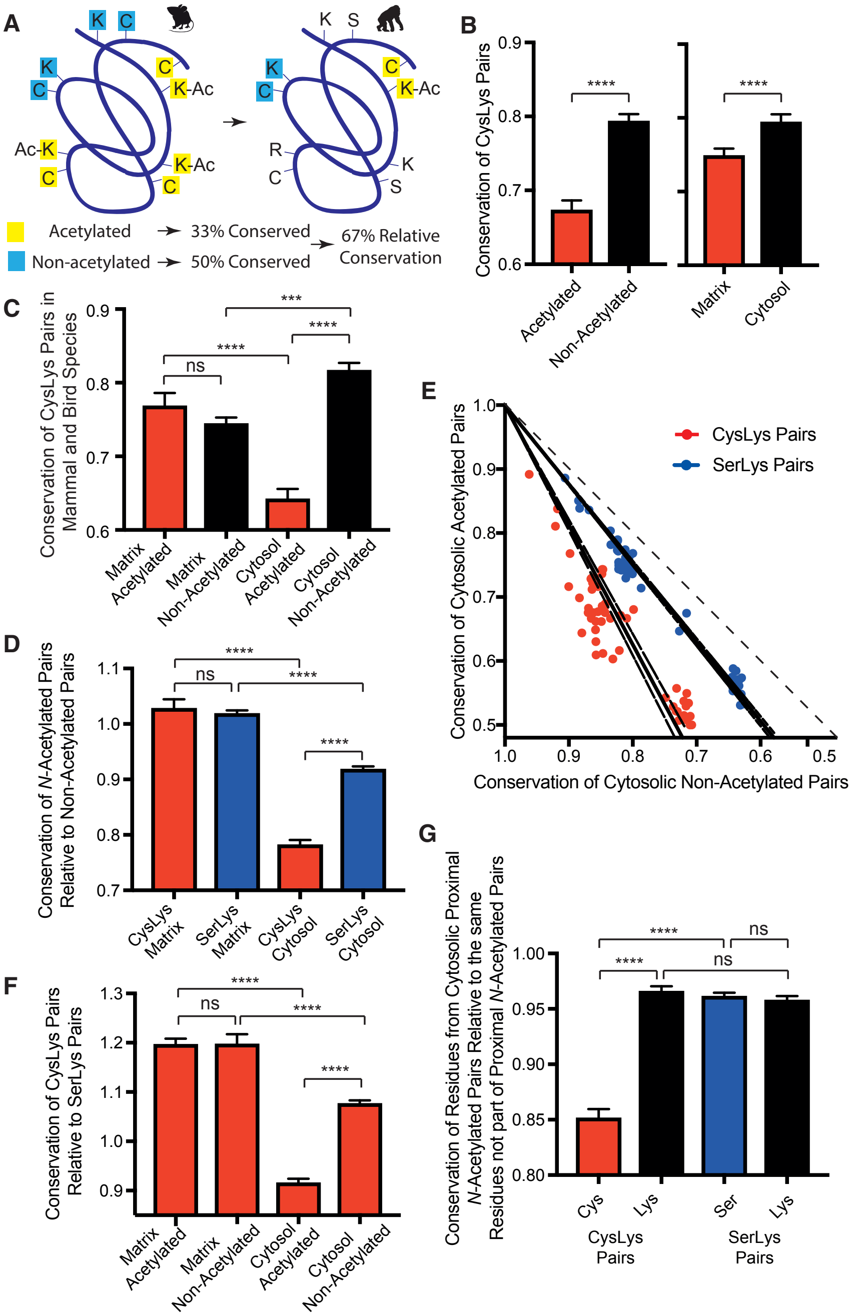

Figure 4. Less Conservation of Proximal *N*-Acetylated CysLys Pairs in 52 Other Mammals and Bird Species

(A) Example of pair conservation.

(B) CysLys pairs are less conserved if they are *N*-acetylated or located in the matrix.

(C) Conservation of cytosolic *N*-acetylated CysLys pairs is less than other CysLys pairs.

(D) *N*-acetylated CysLys pairs are less conserved than non-acetylated CysLys pairs in the cytosol. Data are the average conservation of *N*-acetylated pairs relative to non-acetylated pairs per species.

(E) Cytosolic *N*-acetylated CysLys pairs are less conserved than non-acetylated CysLys pairs. Data are the conservation of *N*-acetylated pairs relative to non-acetylated pairs in each species with linear regression lines and 95% confidence intervals. The line at 45° indicates where values should lie if conservation of *N*-acetylated and non-acetylated pairs was similar.

(F) Cytosolic *N*-acetylated CysLys pairs are less conserved than SerLys pairs. Data are average conservation of *N*-acetylated CysLys pair relative to *N*-acetylated SerLys pairs.

(G) Cytosolic cysteines from *N*-acetylated CysLys pairs are less conserved than other surface cysteines. Data are the average cysteine, lysine, or serine conservation from cytosolic *N*-acetylated pairs relative to all other cytosolic surface cysteines, lysines, or serines not part of these *N*-acetylated pairs.

Data are the mean ± SEM. ***$p < 0.001$; ****$p < 0.0001$.

cytosolic *N*-acetylated CysLys pairs relative to cytosolic *N*-acetylated SerLys pairs is less than one (Figure 4F). This does not arise from differences in the mutation rate between mitochondrial and nuclear genomes, as all analyzed proteins are encoded by the nuclear genome.

This suggests, within vertebrates, cytosolic CysLys pairs are tolerated unless they catalyze $S \rightarrow N$-acyl transfer reactions. In contrast, the acetylating environment of the matrix may have meant readily mutable lysines and CysLys pairs were early targets during the evolution of eukaryotes (Figure 3), and change is difficult to observe within vertebrates (Figure 4).

Cysteines of *N*-Acetylated Cytosolic CysLys Pairs Are Less Conserved

These differences could arise from mutation of either the cysteine, the lysine, or both residues of an *N*-acetylated CysLys pair. Thus, conservation of each cysteine and lysine of a proximal *N*-acetylated CysLys pair (≤11.5Å) was considered independently (Table S4; Figure 4G). Cysteines that were part of proximal *N*-acetylated CysLys pairs in the cytosol were less conserved than cytosolic cysteines not part of proximal *N*-acetylated CysLys pairs. This was not observed for lysines that were part of proximal *N*-acetylated CysLys pairs in the cytosol. It was also not observed for serines or lysines that were part of proximal *N*-acetylated SerLys pairs. As these groups were determined by lysine *N*-acetylation, decreased cysteine conservation provides extra support for an interaction between proximal cysteines and lysines *in vivo*.

Conservation of *N*-Acetylated CysLys Pairs on Cytosolic Proteins Inversely Correlates with Lifespan

Decreased conservation of proximal *N*-acetylated CysLys pairs suggests that lysine *N*-acetylation is detrimental. As dietary restriction and enzymes that could affect cytosolic lysine

OPEN
ACCESS
CellPress

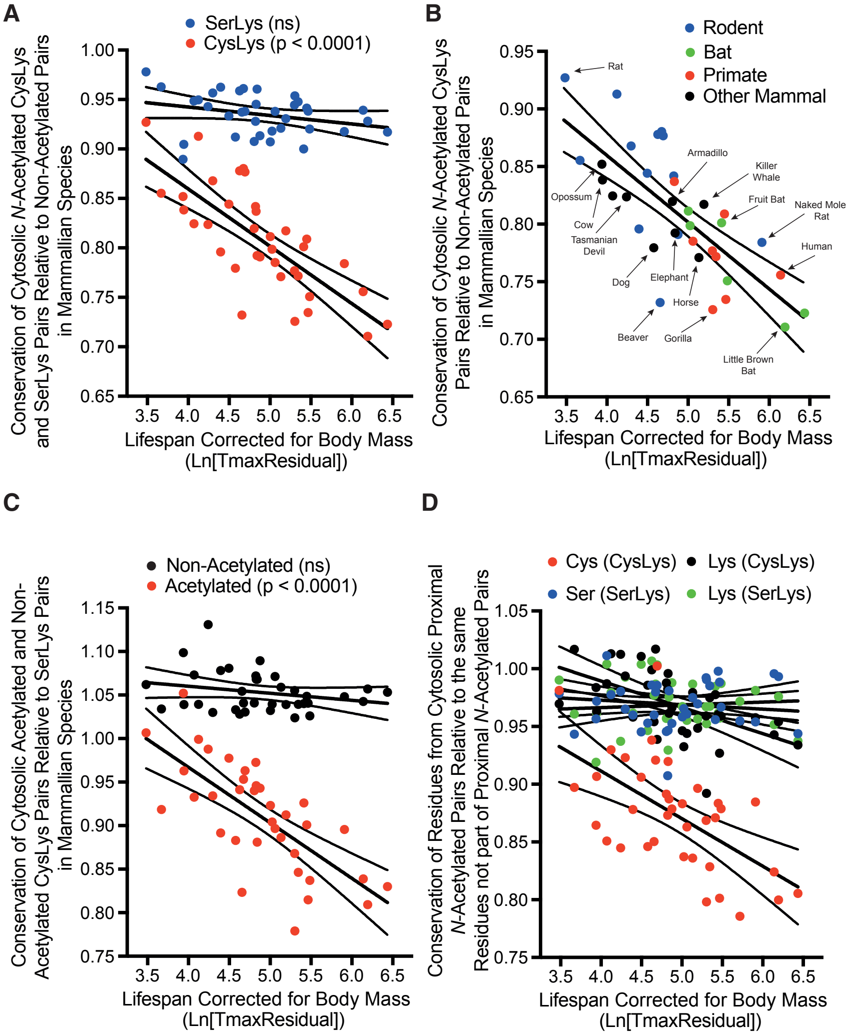

Figure 5. Lifespan Negatively Correlates with Conservation of Proximal Cytosolic *N*-Acetylated CysLys Pairs in 36 Other Mammalian Species

T_{max}Residual is the maximum lifespan of a species as a fraction of the maximum lifespan expected for a mammal of its body mass.

(A) Cytosolic *N*-acetylated CysLys pair conservation negatively correlates with T_{max}Residual in mammals. Data are the conservation of cytosolic *N*-acetylated CysLys (red) and SerLys (blue) pairs relative to non-acetylated pairs.

(B) Phylogeny does not explain the correlation between cytosolic *N*-acetylated CysLys pair conservation and T_{max}Residual. Data are *N*-acetylated CysLys pair conservation in rodents (blue), bats (green), primates (red), and other mammals (black) relative to non-acetylated pairs. Common names are indicated with scientific names in Table S2.

(C) Conservation of cytosolic *N*-acetylated CysLys pairs when expressed relative to *N*-acetylated SerLys pairs negatively correlates with T_{max}Residual. Data are conservation of *N*-acetylated (red) and non-acetylated (black) CysLys pairs relative to SerLys pairs.

(D) Conservation of cytosolic cysteine and lysines that are part of *N*-acetylated proximal CysLys pairs negatively correlates with T_{max}Residual. Proximal cytosolic *N*-acetylated pairs were separated into constituent residues, and conservation of cysteines (red) and lysines (black) part of *N*-acetylated CysLys pairs and serines (blue) and lysines (green) part of *N*-acetylated SerLys pairs was considered independently relative to all other cysteines, serines, or lysines in the cytosol. Lines of best fit are linear regression lines with 95% confidence intervals.

N-acylation are associated with changes in lifespan (Kanfi et al., 2012; Lin et al., 2000; Peleg et al., 2016), we hypothesized that longer-lived animals might favor the loss of proximal CysLys pairs if the lysine can be *N*-acetylated. When conservation of *N*-acetylated CysLys pairs relative to non-acetylated CysLys pairs was plotted against the maximum lifespan of 52 mammal and bird species, there was a highly significant negative correlation ($p < 0.0001$) and no significant correlation for *N*-acetylated cytosolic SerLys pairs ($p = 0.32$; Figure S3A). Body mass is a confounding variable when using maximum lifespan, because larger animals generally live longer (Tacutu et al., 2013; Ma and Gladyshev, 2017). Despite no correlation of weight with *N*-acetylated CysLys pair conservation ($p = 0.96$; Figure S3B), it was desirable to investigate longevity independently of body mass. To correct for body mass, we used T_{max}Residual, which is the observed maximum lifespan of a species as a fraction of the maximum lifespan expected for a mammal of that body mass (Tacutu et al., 2013; Ma and Gladyshev, 2017). When ln(T_{max}Residual) is plotted against cytosolic *N*-acetylated CysLys pair conservation relative to cytosolic non-acetylated CysLys pair conservation, a highly significant negative correlation is evident ($p < 0.0001$; Figure S3C), and this is diminished for cytosolic *N*-acetylated SerLys pairs ($p = 0.014$; Figure S3C). T_{max}Residual was developed for mammals, and the correlation is even clearer if just mammals are considered (Figure 5A). This trend is not significant for SerLys pairs in the cytosol (Figure 5A) or for CysLys and SerLys pairs in the mitochondrial matrix (Figure S3D). Nor is it easily explained by phylogeny as mouse clusters with short-lived marsupials from which it diverged ~160 million years ago, yet is distant from long-lived bats and primates with which it last shared ancestors ~80–100 million years ago (Figures 5B and S3E). This correlation is not caused by clustering of species closely related to mouse as it remains if rodents are excluded and just distant mammalian relatives are considered (Figure S3E). Furthermore, individual mammalian orders show similar correlations (Figure S3F), and the correlation in Figure 5A remains highly significant ($p = 0.0009$) after correction with phylogenetic generalized least-squares (PGLSs) (Figure S4; Table S5).

So far, genomic divergence of *N*-acetylated CysLys pairs has been controlled for by comparing it with conservation of CysLys pairs on the surface of the modeled subunits, where no *N*-acetylated peptide was observed by MS. However, there are caveats

to this, as non-acetylated pairs could be *N*-acetylated and not detected or buried within the quaternary structure *in vivo*. Thus, genomic divergence was also controlled for by expressing conservation of *N*-acetylated CysLys pairs relative to *N*-acetylated SerLys pairs so all lysines are exposed to acetyl-CoA *in vivo* and have been experimentally observed as *N*-acetylated by MS (Weinert et al., 2015). Again, conservation of cytosolic *N*-acetylated CysLys pairs is lower in species with a longer than expected lifespan, and this correlation is highly significant (Figure 5C; p < 0.0001). This correlation is not observed with non-acetylated CysLys pairs (Figure 5C) or matrix *N*-acetylated CysLys pairs (Figure S5A), and it is not explained by phylogeny (Figures S5B–S5D). The correlation in Figure 5C remains highly significant after PGLS (p = 0.0019; Figure S4; Table S5). Thus, the trend arises from *N*-acetylated CysLys pairs and not the procedure used to control for genomic divergence.

Finally, we assessed whether the correlation of conservation of proximal acetylated CysLys pairs in the cytosol with lifespan was caused by their cysteine or lysines (Figure 5D). There was a significant correlation between ln(T_{max}Residual) and conservation of cysteines that were part of proximal *N*-acetylated CysLys pairs relative to all other cysteines (p = 0.0002). This correlation was also significant for lysines from proximal *N*-acetylated CysLys pairs (p = 0.0005) but absent from serines and lysines from proximal *N*-acetylated SerLys pairs (Figure 5D). The correlations in Figure 5D remain significant after PGLS (Figure S4; Table S5).

In summary, both lifespan and lifespan corrected for body mass correlate with the conservation of proximal *N*-acetylated cytosolic CysLys pairs. This is not observed with SerLys pairs, non-acetylated CysLys pairs, or CysLys pairs in the mitochondrial matrix.

DISCUSSION

Lysine *N*-acetylation by acetyl-CoA can be prevented by changing a proximal cysteine to a serine on a peptide or protein (James et al., 2017; Cohen et al., 2013). By generating a 3D structural dataset of mouse proteins previously observed to be *N*-acetylated in mouse liver (Weinert et al., 2015), we show the degree of lysine *N*-acetylation correlates with proximity to a cysteine *in vivo* (Figure 1). That migration of acyl groups from cysteine to proximal lysines occurs *in vivo* is further supported by genomic adaptations between cellular compartments (Figures 3 and 4). In particular, cytosolic proximal *N*-acetylated CysLys pairs were less conserved (Figures 2D and 4), and this primarily resulted from lower cysteine conservation (Figure 4G). As CysLys pairs were grouped by lysine *N*-acetylation, cysteine evolution and lysine *N*-acylation are dependent events. Together, these results indicate that $S \rightarrow N$ transfer reactions are a feature of protein surfaces *in vivo*. Although CysLys pairs were identified using an acetylation dataset, other acyl-CoAs would react with the same pairs, as might reactive dicarbonyls, such as methylglyoxal (Schwarzenbolz et al., 2008).

Average acetylation of lysines with cysteines $\leq$11.5 Å away is 86% higher (p = 0.0002) than other *N*-acetylated lysines. Consequently, each of these lysines contributes disproportionately to the *N*-acetylation load *in vivo*. However, only 5.7% of *N*-acetylated lysines have an intramolecular cysteine $\leq$11.5 Å away. Thus, we estimate proximal cysteines account for 10.7% of the total *N*-acetylation load that occurs on liver proteins *in vivo*, with other mechanisms, such as association of acetyl-CoA with the surface of proteins (Tsuchiya et al., 2017), intermolecular $S \rightarrow S \rightarrow N$-acetyl transfer reactions, and direct *N*-acetylation, also contributing to acetylation load. The relative importance of each of these mechanisms will differ in each cellular compartment and tissue. For example, only the $S \rightarrow S \rightarrow N$-acyl transfer reaction is sensitive to glutathione (GSH) and hydroxyacyl glutathione hydrolase (HAGH) and liver has a particularly high GSH concentration relative to other tissues (James et al., 2017). As the proteomic study used here is from liver and not a post-mitotic tissue often associated with aging, this may also explain the small contribution of the $S \rightarrow S \rightarrow N$-acetyl transfer reaction to *N*-acetylation load despite the strong correlation of proximal *N*-acetylated CysLys pair conservation with lifespan. Even so, our results imply low stoichiometry lysine *N*-acetylation across a range of sites has a functional impact large enough to affect genome-wide changes (Figures 3 and 4).

Exactly why sites of lysine *N*-acetylation are less conserved is beyond the scope of this paper. However, the lack of conservation of a large number of CysLys pairs and the generally low stoichiometry suggest the impact of lysine *N*-acylation is cumulative across a range of sites (Wagner and Hirschey, 2014; Weinert et al., 2015; Trub and Hirschey, 2018). This is supported by recent work showing sirtuin deacetylases suppress acetylation below baseline at a range of sites (Weinert et al., 2017). One potential mechanism is aberrant proteostasis, as autophagy is often required for lifespan extension (Nakamura and Yoshimori, 2018) and lysine *N*-acylation removes ubiquitination sites and positive charges from the surface of a protein, which could increase protein aggregation (Kuczyńska-Wiśnik et al., 2016). Supporting this, *N*-acetyllysine-binding bromodomain proteins are involved in protein aggregation (Olzscha et al., 2017); *N*-acetylated inclusion bodies are larger and harder to resolve (Kuczyńska-Wiśnik et al., 2016); tracts of glutamine, an *N*-acetyl lysine mimetic, lead to aggregation of proteins, such as Huntingtin; and *N*-acetylation of a lysine via a cysteine leads to pathological aggregation of Tau protein (Cohen et al., 2011, 2013).

Such aberrant proteostasis could also explain differences between compartments, as proteins in each are exposed to different acyl-CoAs and aldehydes and have distinct mechanisms of turnover (Green and Levine, 2014; Nakamura and Yoshimori, 2018). That it is conservation of cytosolic *N*-acetylated CysLys pairs that correlates with maximum lifespan (Figure 5) is consistent with genetic interventions that increase longevity. Sir2, a cytosolic deacetylase, is required for dietary restriction to extend lifespan in yeast (Lin et al., 2000), and its mammalian homolog, Sirt1, extends lifespan when overexpressed in the brain (Satoh et al., 2013). Overexpression of Sirt6, a cytosolic long-chain deacylase, extends lifespan in mice (Kanfi et al., 2012), as does knockdown of ATP-citrate lyase, which generates cytosolic acetyl-CoA and thus other acyl-CoAs in flies (Peleg et al., 2016). We note that maximum lifespan is the longest that any individual from a species has been recorded to live, e.g., 122.5 years for humans (Table S2), and it is likely this individual led a relatively healthy lifestyle. Thus, matrix CysLys pairs may

be relevant in pathological settings, such as metabolic syndrome, where overnutrition has been linked to the mitochondrial isoform, Sirt3 (Hirschey et al., 2011; McDonnell et al., 2015).

In summary, we have created a 3D library of acetylated proteins from an existing dataset of mouse liver peptides containing *N*-acetylated lysines (Weinert et al., 2015). This library shows that the enhancement of lysine *N*-acetylation by proximal cysteines occurs at a range of sites *in vivo* (Figure 1). Furthermore, proximal CysLys pairs are less conserved if they can be *N*-acetylated (Figures 2, 3, and 4), and their degree of conservation on cytosolic proteins correlates with maximal lifespan in a large dataset of 52 species each with ~500 proximal CysLys pairs (Figure 5). Which *N*-acyl lysine modifications exert the most selective pressure, which cellular processes and tissues are most affected, and whether lysine *N*-acyl modifications have a causative role in maximum lifespan or other degenerative pathologies remain to be elucidated.

EXPERIMENTAL PROCEDURES

Creation of Mouse Structural Models

A list of *N*-acetylated peptides from mouse liver tissue (Weinert et al., 2015) was used to generate structural models of mouse proteins *N*-acetylated *in vivo*. This and other methodology is described in greater detail in Supplemental Information.

Statistics and Data Processing

Statistical significance was determined using a two-tailed Student's t test or one-way ANOVA followed by a Dunnett's multiple comparison test. Differences in frequency were tested using two-sided chi-square tests. For linear regression, lines are displayed with 95% confidence intervals. Where indicated, p values were corrected for phylogenetic bias using the PGLS method with Pagel's λ to estimate phylogenetic signal.

SUPPLEMENTAL INFORMATION

Supplemental Information includes Supplemental Experimental Procedures, five figures, and five tables and can be found with this article online at https://doi.org/10.1016/j.celrep.2018.07.007.

ACKNOWLEDGMENTS

This work was supported by the Medical Research Council UK (MC_U105663142 to M.P.M.; MC_U105674181 to A.J.R.) and by a Wellcome Trust Investigator award (110159/Z/15/Z) to M.P.M.

AUTHOR CONTRIBUTIONS

Conceptualization, A.M.J. and M.P.M.; Methodology, A.M.J., C.L.S., and A.C.S.; Software, A.C.S., C.L.S., and A.J.R.; Formal Analysis, A.M.J. and C.L.S.; Writing – Original Draft, A.M.J.; Writing – Review and Editing, A.M.J., C.L.S., M.P.M., A.C.S., and A.J.R.; Project Administration, A.M.J.; Supervision, M.P.M. and A.J.R.; Funding Acquisition, M.P.M. and A.J.R.

DECLARATION OF INTERESTS

The authors declare no competing interests.

Received: December 14, 2017
Revised: April 19, 2018
Accepted: July 1, 2018
Published: August 7, 2018

REFERENCES

Baeza, J., Smallegan, M.J., and Denu, J.M. (2016). Mechanisms and dynamics of protein acetylation in mitochondria. Trends Biochem. Sci. *41*, 231–244.

Bizzozero, O.A., Bixler, H.A., and Pastuszyn, A. (2001). Structural determinants influencing the reaction of cysteine-containing peptides with palmitoyl-coenzyme A and other thioesters. Biochim. Biophys. Acta *1545*, 278–288.

Cohen, T.J., Guo, J.L., Hurtado, D.E., Kwong, L.K., Mills, I.P., Trojanowski, J.Q., and Lee, V.M. (2011). The acetylation of tau inhibits its function and promotes pathological tau aggregation. Nat. Commun. *2*, 252.

Cohen, T.J., Friedmann, D., Hwang, A.W., Marmorstein, R., and Lee, V.M. (2013). The microtubule-associated tau protein has intrinsic acetyltransferase activity. Nat. Struct. Mol. Biol. *20*, 756–762.

Fiser, A., and Sali, A. (2003). Modeller: generation and refinement of homology-based protein structure models. Methods Enzymol. *374*, 461–491.

Gould, N.S., Evans, P., Martínez-Acedo, P., Marino, S.M., Gladyshev, V.N., Carroll, K.S., and Ischiropoulos, H. (2015). Site-specific proteomic mapping identifies selectively modified regulatory cysteine residues in functionally distinct protein networks. Chem. Biol. *22*, 965–975.

Green, D.R., and Levine, B. (2014). To be or not to be? How selective autophagy and cell death govern cell fate. Cell *157*, 65–75.

Hirschey, M.D., Shimazu, T., Jing, E., Grueter, C.A., Collins, A.M., Aouizerat, B., Stančáková, A., Goetzman, E., Lam, M.M., Schwer, B., et al. (2011). SIRT3 deficiency and mitochondrial protein hyperacetylation accelerate the development of the metabolic syndrome. Mol. Cell *44*, 177–190.

James, A.M., Hoogewijs, K., Logan, A., Hall, A.R., Ding, S., Fearnley, I.M., and Murphy, M.P. (2017). Non-enzymatic n-acetylation of lysine residues by acetylCoA often occurs via a proximal s-acetylated thiol intermediate sensitive to glyoxalase ii. Cell Rep. *18*, 2105–2112.

Kanfi, Y., Naiman, S., Amir, G., Peshti, V., Zinman, G., Nahum, L., Bar-Joseph, Z., and Cohen, H.Y. (2012). The sirtuin SIRT6 regulates lifespan in male mice. Nature *483*, 218–221.

Kuczyńska-Wiśnik, D., Moruno-Algara, M., Stojowska-Swędrzyńska, K., and Laskowska, E. (2016). The effect of protein acetylation on the formation and processing of inclusion bodies and endogenous protein aggregates in Escherichia coli cells. Microb. Cell Fact. *15*, 189.

Lin, S.J., Defossez, P.A., and Guarente, L. (2000). Requirement of NAD and SIR2 for life-span extension by calorie restriction in Saccharomyces cerevisiae. Science *289*, 2126–2128.

Ma, S., and Gladyshev, V.N. (2017). Molecular signatures of longevity: insights from cross-species comparative studies. Semin. Cell Dev. Biol. *70*, 190–203.

McDonnell, E., Peterson, B.S., Bomze, H.M., and Hirschey, M.D. (2015). SIRT3 regulates progression and development of diseases of aging. Trends Endocrinol. Metab. *26*, 486–492.

Nakamura, S., and Yoshimori, T. (2018). Autophagy and longevity. Mol. Cells *41*, 65–72.

Olzscha, H., Fedorov, O., Kessler, B.M., Knapp, S., and La Thangue, N.B. (2017). Cbp/p300 bromodomains regulate amyloid-like protein aggregation upon aberrant lysine acetylation. Cell Chem. Biol. *24*, 9–23.

Pan, H., and Finkel, T. (2017). Key proteins and pathways that regulate lifespan. J. Biol. Chem. *292*, 6452–6460.

Peleg, S., Feller, C., Forne, I., Schiller, E., Sévin, D.C., Schauer, T., Regnard, C., Straub, T., Prestel, M., Klima, C., et al. (2016). Life span extension by targeting a link between metabolism and histone acetylation in Drosophila. EMBO Rep. *17*, 455–469.

Peng, C., Lu, Z., Xie, Z., Cheng, Z., Chen, Y., Tan, M., Luo, H., Zhang, Y., He, W., Yang, K., et al. (2011). The first identification of lysine malonylation substrates and its regulatory enzyme. Mol. Cell Proteomics *10*, M111.012658.

Pietrocola, F., Galluzzi, L., Bravo-San Pedro, J.M., Madeo, F., and Kroemer, G. (2015). Acetyl coenzyme A: a central metabolite and second messenger. Cell Metab. *21*, 805–821.

Rardin, M.J., Newman, J.C., Held, J.M., Cusack, M.P., Sorensen, D.J., Li, B., Schilling, B., Mooney, S.D., Kahn, C.R., Verdin, E., and Gibson, B.W. (2013). Label-free quantitative proteomics of the lysine acetylome in mitochondria identifies substrates of SIRT3 in metabolic pathways. Proc. Natl. Acad. Sci. USA *110*, 6601–6606.

Requejo, R., Hurd, T.R., Costa, N.J., and Murphy, M.P. (2010). Cysteine residues exposed on protein surfaces are the dominant intramitochondrial thiol and may protect against oxidative damage. FEBS J. *277*, 1465–1480.

Satoh, A., Brace, C.S., Rensing, N., Cliften, P., Wozniak, D.F., Herzog, E.D., Yamada, K.A., and Imai, S. (2013). Sirt1 extends life span and delays aging in mice through the regulation of Nk2 homeobox 1 in the DMH and LH. Cell Metab. *18*, 416–430.

Schwarzenbolz, U., Mende, S., and Henle, T. (2008). Model studies on protein glycation: influence of cysteine on the reactivity of arginine and lysine residues toward glyoxal. Ann. N Y Acad. Sci. *1126*, 248–252.

Tacutu, R., Craig, T., Budovsky, A., Wuttke, D., Lehmann, G., Taranukha, D., Costa, J., Fraifeld, V.E., and de Magalhães, J.P. (2013). Human Ageing Genomic Resources: integrated databases and tools for the biology and genetics of ageing. Nucleic Acids Res. *41*, D1027–D1033.

Tan, M., Peng, C., Anderson, K.A., Chhoy, P., Xie, Z., Dai, L., Park, J., Chen, Y., Huang, H., Zhang, Y., et al. (2014). Lysine glutarylation is a protein posttranslational modification regulated by SIRT5. Cell Metab. *19*, 605–617.

Trub, A.G., and Hirschey, M.D. (2018). Reactive acyl-coa species modify proteins and induce carbon stress. Trends Biochem. Sci. *43*, 369–379.

Tsuchiya, Y., Peak-Chew, S.Y., Newell, C., Miller-Aidoo, S., Mangal, S., Zhyvoloup, A., Bakovic, J., Malanchuk, O., Pereira, G.C., Kotiadis, V., et al. (2017). Protein CoAlation: a redox-regulated protein modification by coenzyme A in mammalian cells. Biochem. J. *474*, 2489–2508.

Wagner, G.R., and Hirschey, M.D. (2014). Nonenzymatic protein acylation as a carbon stress regulated by sirtuin deacylases. Mol. Cell *54*, 5–16.

Wagner, G.R., and Payne, R.M. (2013). Widespread and enzyme-independent Nε-acetylation and Nε-succinylation of proteins in the chemical conditions of the mitochondrial matrix. J. Biol. Chem. *288*, 29036–29045.

Weinert, B.T., Schölz, C., Wagner, S.A., Iesmantavicius, V., Su, D., Daniel, J.A., and Choudhary, C. (2013). Lysine succinylation is a frequently occurring modification in prokaryotes and eukaryotes and extensively overlaps with acetylation. Cell Rep. *4*, 842–851.

Weinert, B.T., Moustafa, T., Iesmantavicius, V., Zechner, R., and Choudhary, C. (2015). Analysis of acetylation stoichiometry suggests that SIRT3 repairs nonenzymatic acetylation lesions. EMBO J. *34*, 2620–2632.

Weinert, B.T., Satpathy, S., Hansen, B.K., Lyon, D., Jensen, L.J., and Choudhary, C. (2017). Accurate quantification of site-specific acetylation stoichiometry reveals the impact of sirtuin deacetylase cobb on the e. Coli acetylome. Mol. Cell. Proteomics *16*, 759–769.

Cell Reports, Volume *24*

Supplemental Information

Proximal Cysteines that Enhance Lysine *N*-Acetylation of Cytosolic Proteins in Mice Are Less Conserved in Longer-Living Species

Andrew M. James, Anthony C. Smith, Cassandra L. Smith, Alan J. Robinson, and Michael P. Murphy

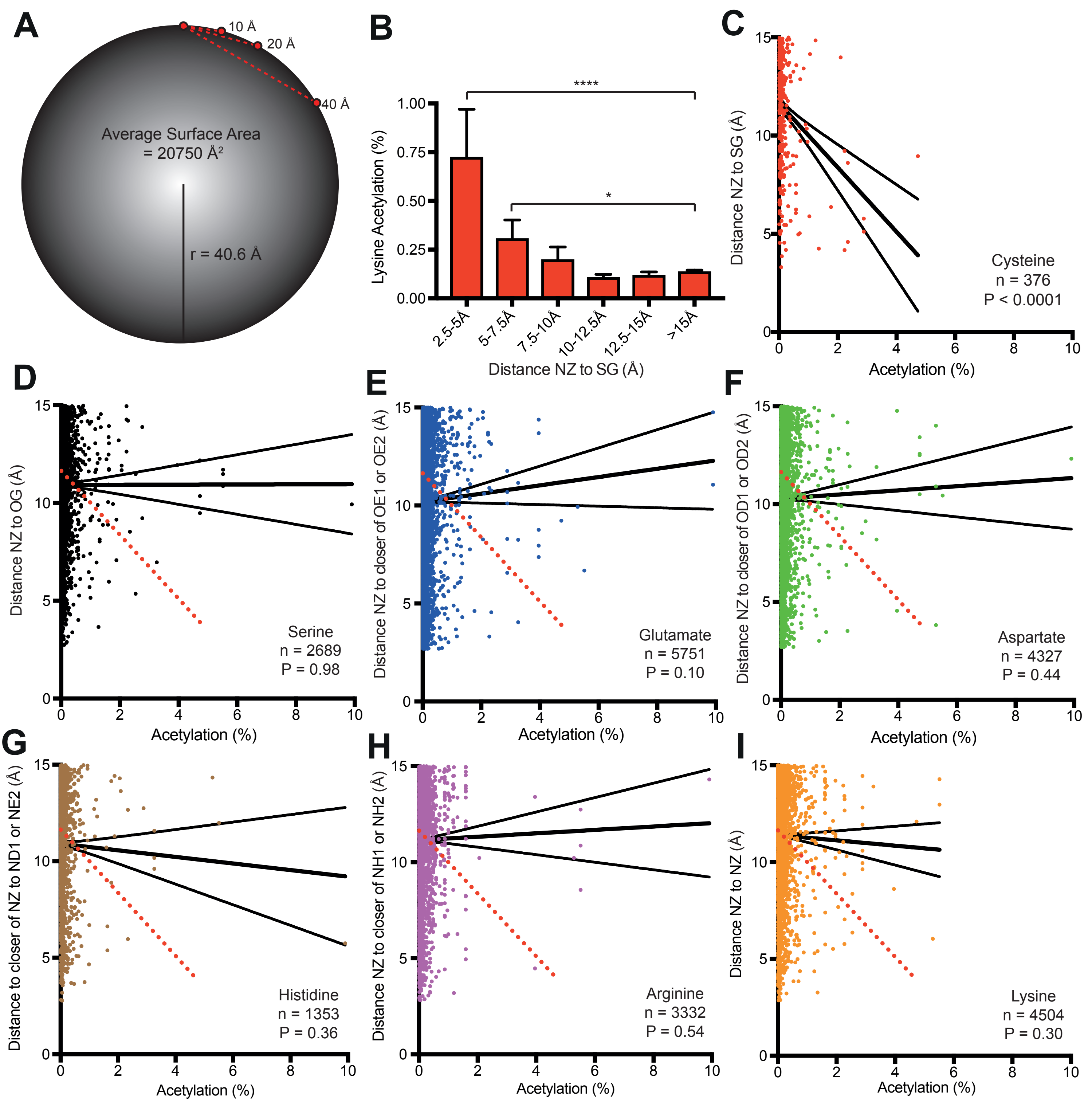

Figure S1. Protein lysine N-acetylation is increased by proximity to a cysteine. Related to Figure 1. The mouse liver dataset of acetylation sites and their degree of lysine N-acetylation is from (Weinert et al., 2015). Mouse homologs of 622 of these acetylated proteins were modelled from existing molecular structures in other species and the distance between their lysine amine (NZ) and a second atom was calculated using trigonometry. Only pairs where >5Å2 of both atoms were exposed on the surface, and where the NZ atom was N-acetylated, were included. A, reactions between residues >~15 Å apart are sterically hindered. The surface area of the 619 proteins was used to calculate their average radius. B, lysine N-acetylation increases with proximity to a cysteine thiol. Only the closest SG atom to each N-acetylated NZ atom was considered and CysLys pairs were grouped by NZ and SG distance. C, lysine N-acetylation negatively correlates with NZ to SG distance. Data are individual SG to NZ distances for pairs of cysteine and N-acetylated lysine residues <15 Å apart. D, lysine N-acetylation does not correlate with distance to a serine residue. Data are individual NZ to OG distances for pairs of serine and N-acetylated lysine residues <15 Å apart. E, lysine N-acetylation does not correlate with distance to a glutamate residue. Data are individual NZ to closest OE1/OE2 distances for pairs of glutamate and N-acetylated lysine residues <15 Å apart. F, lysine N-acetylation does not correlate with distance to an aspartate residue. Data are individual NZ to closest OD1/OD2 distances for pairs of aspartate and N-acetylated lysine residues <15 Å apart. G, lysine N-acetylation does not correlate with distance to a histidine residue. Data are individual NZ to closest ND1/NE2 distances for pairs of histidine and N-acetylated lysine residues <15 Å apart. H, lysine N-acetylation does not correlate with distance to an arginine residue. Data are individual NZ to closest NH1/NH2 distances for pairs of arginine and N-acetylated lysine residues <15 Å apart. I, lysine N-acetylation does not correlate with distance to another lysine residue. Data are individual NZ to NZ distances for pairs of lysine and N-acetylated lysine residues <15 Å apart. Solid black lines are linear regression lines of the points on each graph with 95% confidence intervals. The red dotted line is the cysteine regression line from Figure S1C.

A

Uniprot	Protein Name	Lys	Cys	Acet	Dist (Å)
Q91VA0	Acyl-coenzyme A synthetase (ACSM1)	200	196	4.73%	8.96
Q8CHR6	Dihydropyrimidine dehydrogenase [$NADP^+$]	384	49	2.89%	5.11
Q8CHR6	Dihydropyrimidine dehydrogenase [$NADP^+$]	384	52	2.89%	5.78
Q9DCM0	Persulfide dioxygenase (ETHE1)	172	170	2.34%	8.61
Q9DCM0	Persulfide dioxygenase (ETHE1)	172	219	2.34%	4.59
O08997	Copper transport protein (ATOX1)	60	12	2.23%	9.22
O08997	Copper transport protein (ATOX1)	60	15	2.23%	4.18

B

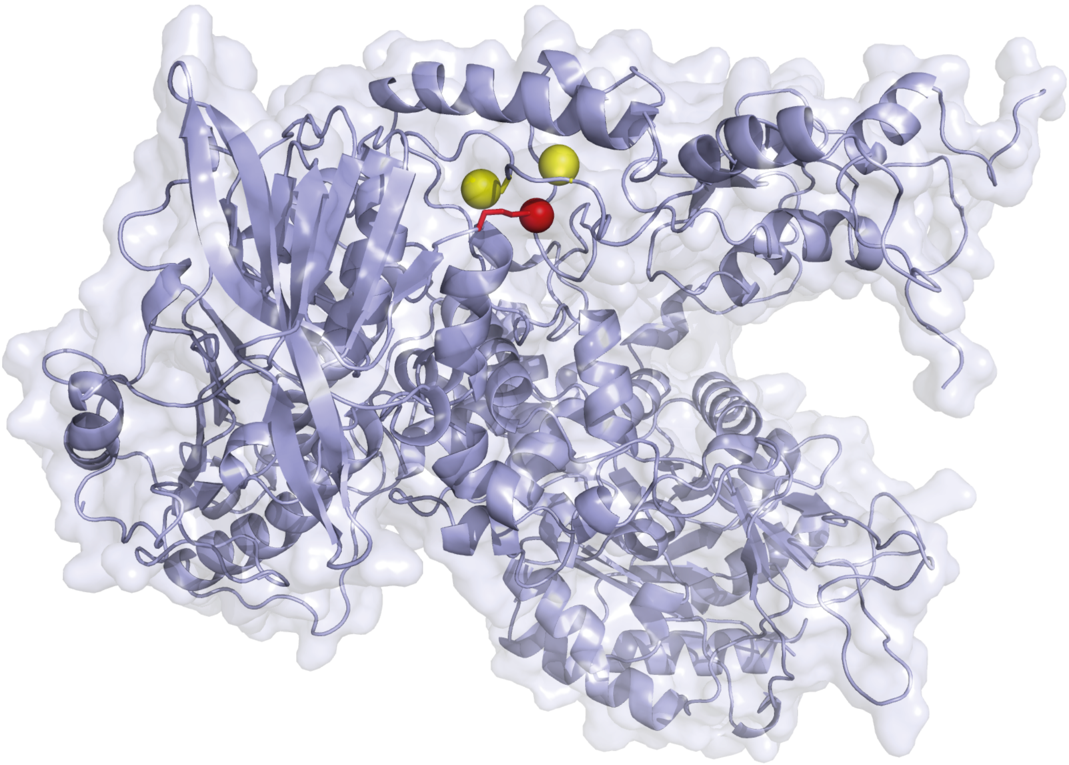

Figure S2. The most N-acetylated CysLys pairs frequently have another nearby cysteine. Related to Figure 2. A, the most N-acetylated lysine residues with a proximal cysteine residue often have additional close cysteine residues. B, a mouse model of pig dihydropyrimidine dehydrogenase (1GTE) contains a lysine residue (red) that can be N-acetylated with two proximal cysteine thiols (yellow).

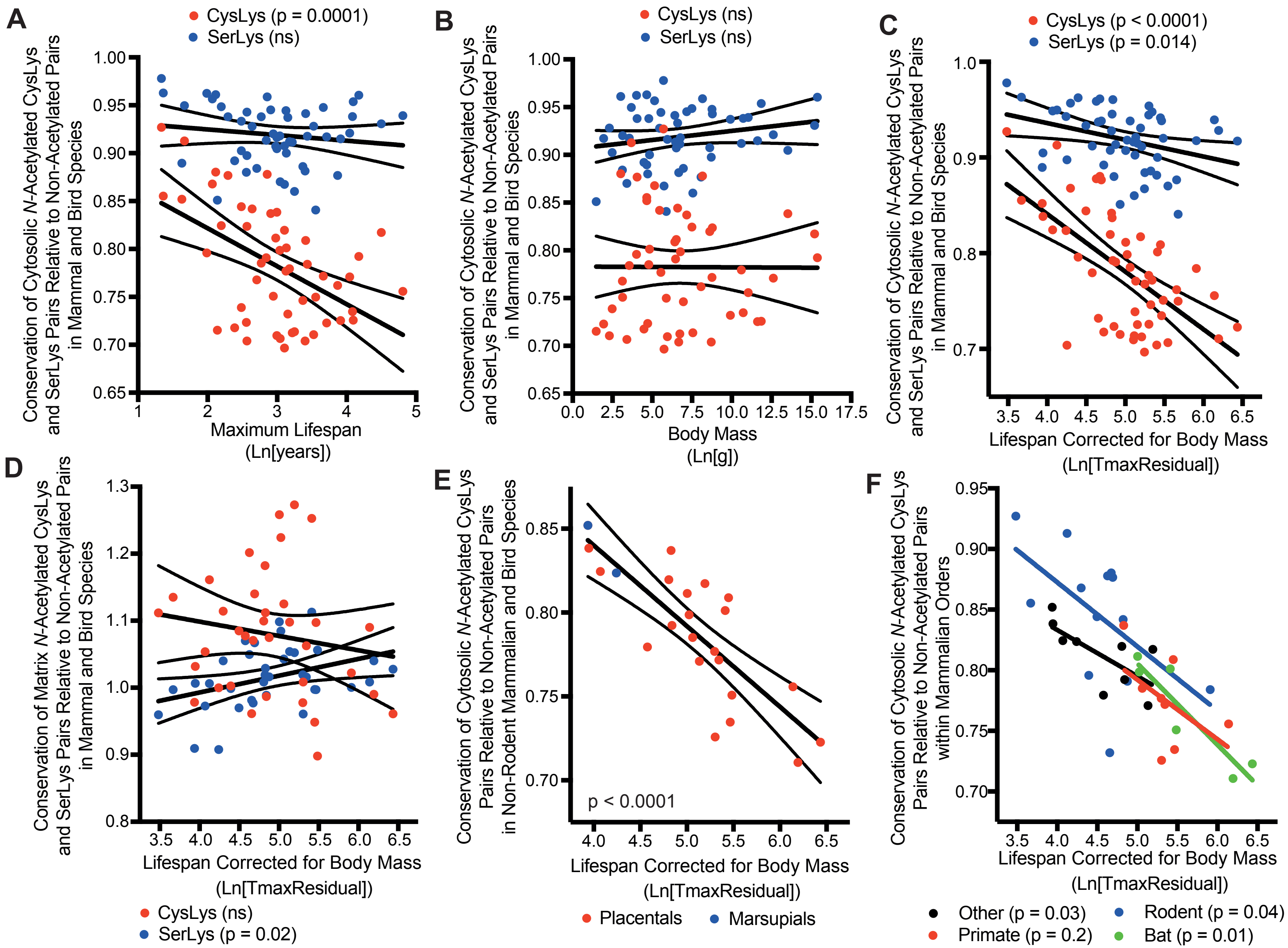

Figure S3. Phylogeny does not explain why conservation N-acetylated CysLys pairs in the cytosol negatively correlates with lifespan. Related to Figure 5. A, conservation of cytosolic N-acetylated CysLys pairs negatively correlates with maximum lifespan in mammals and birds. Data is the conservation of N-acetylated CysLys (red) and SerLys (blue) pairs relative to non-acetylated pairs in 52 mammal and bird species. B, conservation of cytosolic N-acetylated CysLys pairs does not correlate with body mass in mammals and birds. Data is the conservation of N-acetylated CysLys (red) and SerLys (blue) pairs relative to non-acetylated pairs in 52 mammal and bird species. C, conservation of cytosolic N-acetylated CysLys pairs negatively correlates with lifespan corrected for body mass (TmaxResidual) in mammals and birds. TmaxResidual is the maximum lifespan for a species as a percentage of the maximum lifespan expected for a mammal of its body mass. Data is the conservation of N-acetylated CysLys (red) and SerLys (blue) pairs relative to non-acetylated pairs in 52 mammal and bird species. D, conservation of N-acetylated CysLys pairs in the mitochondrial matrix does not correlate with TmaxResidual. Data is the conservation of N-acetylated CysLys (red) and SerLys (blue) pairs relative to non-acetylated pairs in 36 mammal species. E, lower cytosolic N-acetylated CysLys pair conservation is not a consequence of evolutionary distance from mouse. Data is the conservation of cytosolic N-acetylated CysLys pairs relative to non-acetylated CysLys pairs in non-rodent placental (red) and marsupial (blue) species that diverged from mouse >~80 million years ago. Rodent species were excluded to ensure their smaller evolutionary distance from mouse did not cause the correlation with TmaxResidual. The genetically distant marsupial species share low selective pressure against cytosolic acetylated CysLys pairs and short lifespans with mouse. For comparison, mouse ln(TmaxResidual) is 3.94 and relative cytosolic acetylated CysLys pair conservation is 1. F, correlation between cytosolic N-acetylated CysLys pair conservation and TmaxResidual in three mammalian orders. Data is the conservation of cytosolic N-acetylated CysLys pairs in rodents (blue), bats (green) and primates (red) relative to non-acetylated pairs. Other mammals that are not rodents, bats or primates are also shown (black). Lines of best fit are their respective linear regression lines and 95% confidence intervals.

A

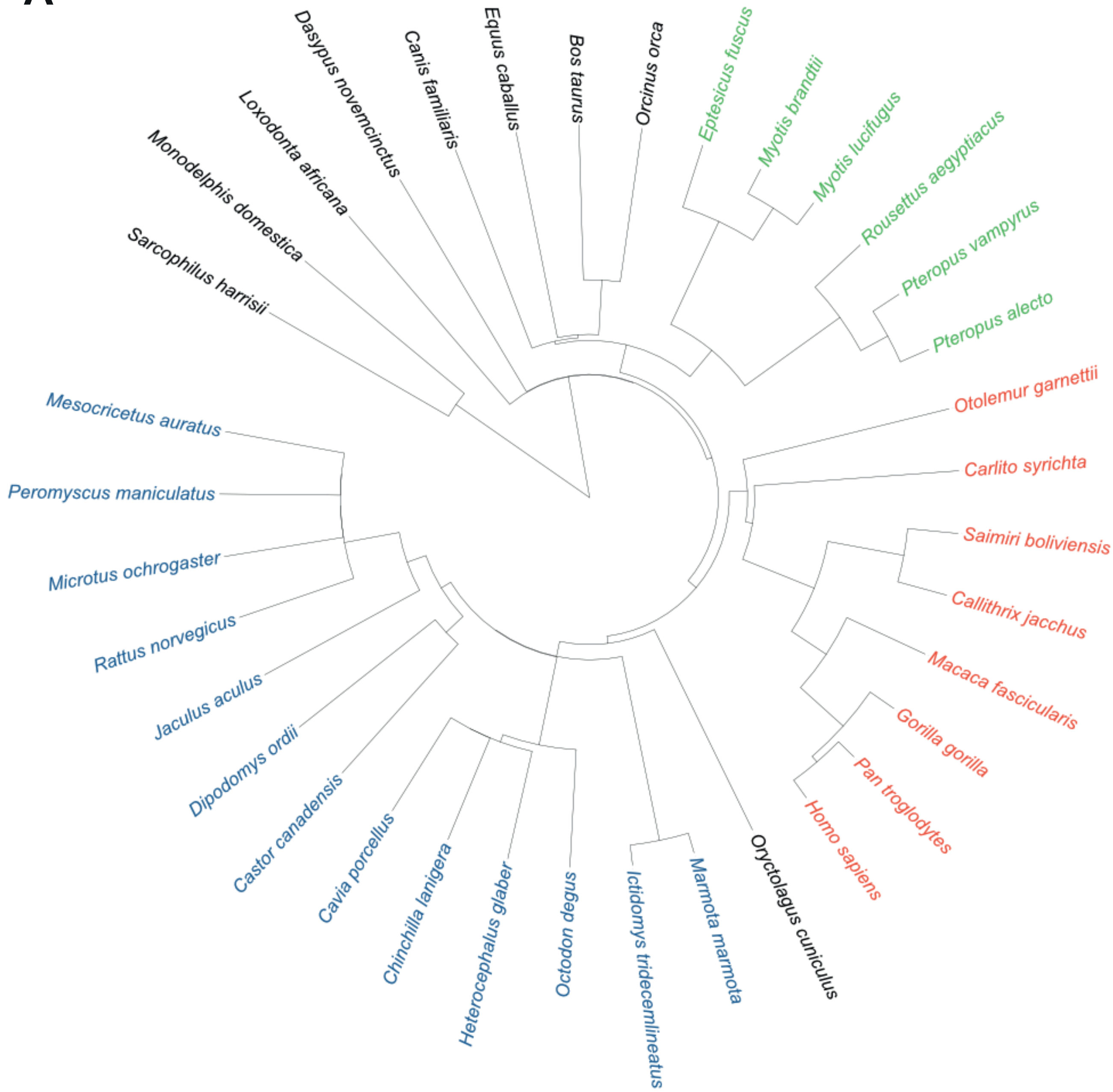

B

	Regression				Phylogenetic Generalized Least Squares (PGLS)		
**Figure 5A**	**y-int**	**slope**	**p-value**	**Pagel's λ**	**y-int**	**slope**	**p-value**
**CysLys**	1.09	-0.058	1.36E-07	0.79	1.00	-0.040	9.00E-04
**SerLys**	0.98	-0.008	0.092	1.02	0.96	-0.006	0.14
**Figure 5C**	**y-int**	**slope**	**p-value**	**Pagel's λ**	**y-int**	**slope**	**p-value**
**Acetylated**	1.22	-0.064	1.18E-06	0.76	1.14	-0.043	0.0019
**Non-acetylated**	1.09	-0.008	0.16	1.05	1.09	0.001	0.72
**Figure 5D**	**y-int**	**slope**	**p-value**	**Pagel's λ**	**y-int**	**slope**	**p-value**
**CysLys - Cys**	1.07	-0.040	2.24E-04	0.81	0.99	-0.027	0.027
**CysLys - Lys**	1.08	-0.023	5.07E-04	0.15	1.02	-0.021	0.0021
**SerLys - Ser**	0.97	-0.003	0.95	0.96	0.96	0.002	0.66
**SerLys - Lys**	0.99	-0.004	0.46	0.82	0.97	-0.003	0.66

Figure S4. Phylogenetic generalised least squares. Related to Figure 5. A, the phylogenetic tree for the phylogenetic generalised least squares (PGLS) method was based on data from the mammalian tree described (Bininda-Emonds et al., 2007). B, the significant correlations of lifespan with cytosolic N-acetylated CysLys pair conservation remain after phylogenetic correction with PGLS. Pagel's λ estimates phylogenetic signal (Pagel, 1999).

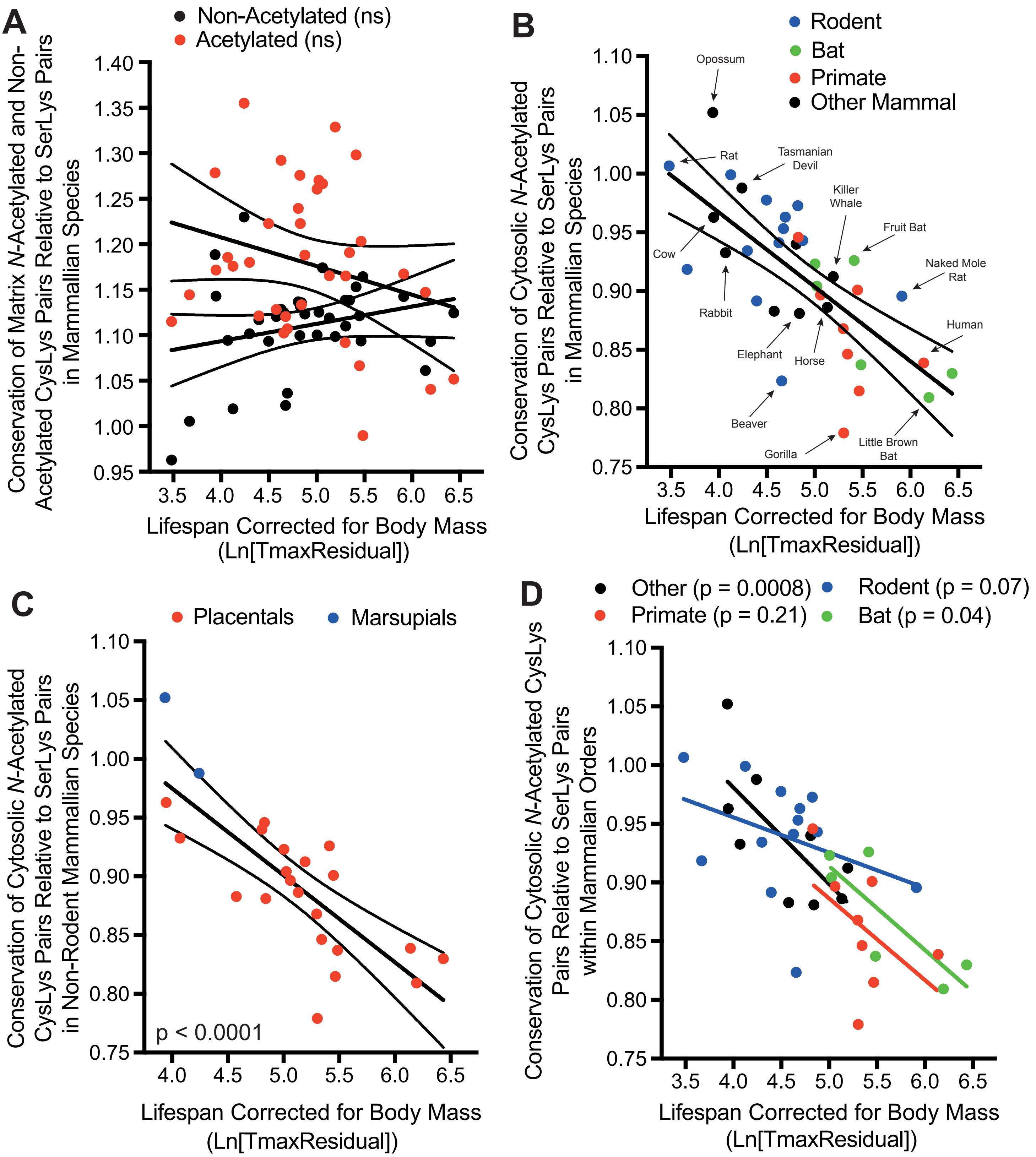

Figure S5. The correlation of lifespan with cytosolic N-acetylated CysLys pair conservation remains when expressed relative to N-acetylated SerLys pairs. Related to Figure 5. TmaxResidual is the maximum lifespan for a species as a percentage of the maximum lifespan expected for a mammal of its body mass. A, conservation of N-acetylated CysLys pairs in the mitochondrial matrix does not correlate with TmaxResidual. Data is the conservation of N-acetylated (red) and non-acetylated (black) CysLys pairs relative to SerLys pairs. B, phylogeny does not explain the correlation between cytosolic N-acetylated CysLys pair conservation and TmaxResidual. Data is N-acetylated CysLys pair conservation in rodents (blue), bats (green), primates (red) and other mammals (black) relative to N-acetylated SerLys pairs. Common names are indicated with scientific names in Table S2. C, lower cytosolic N-acetylated CysLys pair conservation is not a consequence of evolutionary distance from mouse. Data is the conservation of cytosolic N-acetylated CysLys pairs relative to N-acetylated SerLys pairs in non-rodent placental (red) and marsupial (blue) species that diverged from mouse >~80 million years ago. Rodent species were excluded to ensure their smaller evolutionary distance from mouse did not cause the correlation with TmaxResidual. The genetically distant marsupial species share low selective pressure against cytosolic acetylated CysLys pairs and short lifespans with mouse. For comparison, mouse ln(TmaxResidual) is 3.94 and relative cytosolic acetylated CysLys pair conservation is 1. D, correlation between cytosolic N-acetylated CysLys pair conservation and TmaxResidual in three mammalian orders. Data is the conservation of cytosolic N-acetylated CysLys pairs in rodents (blue), bats (green) and primates (red) relative to N-acetylated SerLys pairs. Other mammals that are not rodents, bats or primates are also shown (black). Lines of best fit are their respective linear regression lines and 95% confidence intervals.

SUPPLEMENTAL EXPERIMENTAL PROCEDURES

Creation of mouse structural models – A list of acetylated proteins from mouse liver tissue was obtained from the literature (Weinert et al., 2015). The corresponding protein sequences were taken from UniProt and individually aligned with those in a non-redundant PDB sequence database clustered at 95%, using MODELLER (Webb and Sali, 2016). The best match that had a minimum sequence identity of 50% across their entire length of the query sequence was then used as a structural template. The query sequence was then structurally aligned with the template and this was used to create five predicted structures, with the one with the lowest DOPE score retained for analysis. For each structure the solvent accessible area in $Å^2$ of every atom was calculated using areaimol from the CCP4 software suite (Winn et al., 2011). Distances between atoms of interest were calculated using trigonometry from the 3D coordinates in the generated PDB files.

Definition of cytosolic and mitochondrial matrix proteins – Matrix proteins were classified as those with Gene Ontology annotation (using both human and mouse annotation), present in large-scale matrix APEX tagging study (Rhee et al., 2013) or defined in the matrix compartment of a metabolic model of the mitochondrion (Smith et al.). Cytosolic proteins were defined as those annotated as such in the Gene Ontology (mouse or human) or had been experimentally determined to be cytosolic in the Human Protein Atlas (Thul et al., 2017) (evidence level: validated or supported). Proteins that were dual localized were removed.

Identification of orthologues - For each of the modelled mouse proteins, human orthologs were identified manually using orthology information from the MitoMiner database (Smith and Robinson, 2016). Proteins with no clear one to one human ortholog were removed from the analysis to reduce interference from paralogous proteins. Orthologs of each human protein were identified in 70 additional vertebrates using a BLASTp search against a local BLASTp database containing non-identical protein sequences of each species, with an E-value cut-off of $1e^{-10}$. The top protein hit for each species was considered orthologous if a reciprocal BLASTp search against a database of all human proteins with an assigned gene name from NCBI returned a protein equivalent to the original human protein as top hit (E-value cut-off of $1e^{-10}$). Top hits for each of the species were used to perform local BLASTp searches against a database of human proteins. It was called an ortholog if the reciprocal search returned a protein equivalent to the original human protein (E-value cut-off $1e^{-10}$). Proteins with less than 60 orthologues were removed from the analysis, leaving 442 proteins. Species outlying numbers of identified orthologs (less than lower quartile-1.5*interquartile range) were also removed, leaving 66 species plus mouse for further analysis.

Conservation of residues and pairs - For each gene, protein sequences of identified orthologues were aligned using MUSCLE (default settings) (Edgar, 2004). For each lysine, cysteine or serine identified as part of a surface (>5% $Å^2$) pair with ≤11.5 Å distance between them in mouse, the aligned residue was identified in each other species. Within each species, for each CysLys and SerLys pair, the pair was conserved if both residues exactly matched mouse, not conserved if either residue did not match mouse, and not present if at least one position had no aligned residue. Overall species conservation was calculated as number of conserved pairs/number of present pairs. Calculations were also made for matrix, cytosol, acetylated and non-acetylated pairs and combinations of these. Single residue conservation was analysed in the same manner. Non-acetylated pairs were those pairs identified as being close in the modelled structure and that were not observed to be acetylated by MS.

Maximum lifespan analysis - Maximum recorded lifespan and weight of species was retrieved from the AnAge database for most species (Tacutu et al., 2013). T_{max}Residual was calculated as (maximum lifespan/(4.88*(adult weight$^{0.153}$)))*100, as in the AnAge database.

Statistics and data processing – Statistical analysis was performed in Prism v6. Statistical significance was determined using a two-tailed Student's t-test or one-way ANOVA followed by a Dunnett's multiple comparison test. For linear regression, lines are displayed with their 95% confidence intervals. P-values for linear regression are the probability that the slope of the regression line is zero and there is no correlation. Differences in frequency were tested using two-sided Chi-square tests.

Phylogenetic generalised least squares - The phylogenetic tree was based on data from the mammalian tree described (Bininda-Emonds et al., 2007). As *Sarcophilus harrisii* was not present in the tree, the position of the closely related *Sarcophilus laniarius* from the same genus was used to represent this species. The phylogenetic generalised least squares method (Grafen, 1989) was implemented using the R package nlme to correct for phylogenetic bias, using Pagel's λ (Pagel, 1999) to estimate phylogenetic signal.

REFERENCES

Bininda-Emonds, O.R., Cardillo, M., Jones, K.E., MacPhee, R.D., Beck, R.M., Grenyer, R., Price, S.A., Vos, R.A., Gittleman, J.L. & Purvis, A. (2007). The delayed rise of present-day mammals. Nature *446*, 507-12.

Edgar, R.C. (2004). Muscle: Multiple sequence alignment with high accuracy and high throughput. Nucleic Acids Res *32*, 1792-7.

Grafen, A. (1989). The phylogenetic regression. Philos Trans R Soc Lond B Biol Sci *326*, 119-57.

Pagel, M. (1999). Inferring the historical patterns of biological evolution. Nature *401*, 877-84.

Rhee, H.W., Zou, P., Udeshi, N.D., Martell, J.D., Mootha, V.K., Carr, S.A. & Ting, A.Y. (2013). Proteomic mapping of mitochondria in living cells via spatially restricted enzymatic tagging. Science *339*, 1328-1331.

Smith, A.C., Eyassu, F., Mazat, J.-P. & Robinson, A.J. Mitocore: A curated constraint-based model for simulating human central metabolism. BMC Systems Biology *In Press*.

Smith, A.C. & Robinson, A.J. (2016). Mitominer v3.1, an update on the mitochondrial proteomics database. Nucleic Acids Res *44*, D1258-61.

Tacutu, R., Craig, T., Budovsky, A., Wuttke, D., Lehmann, G., Taranukha, D., Costa, J., Fraifeld, V.E. & de Magalhaes, J.P. (2013). Human ageing genomic resources: Integrated databases and tools for the biology and genetics of ageing. Nucleic Acids Res *41*, D1027-33.

Thul, P.J., Akesson, L., Wiking, M., Mahdessian, D., Geladaki, A., Ait Blal, H., Alm, T., Asplund, A., Bjork, L., Breckels, L.M., et al. (2017). A subcellular map of the human proteome. Science *356*.

Webb, B. & Sali, A. (2016). Comparative protein structure modeling using modeller. Curr Protoc Protein Sci *86*, 2.9.1-2.9.37.

Weinert, B.T., Moustafa, T., Iesmantavicius, V., Zechner, R. & Choudhary, C. (2015). Analysis of acetylation stoichiometry suggests that sirt3 repairs nonenzymatic acetylation lesions. EMBO J *34*, 2620-32.

Winn, M.D., Ballard, C.C., Cowtan, K.D., Dodson, E.J., Emsley, P., Evans, P.R., Keegan, R.M., Krissinel, E.B., Leslie, A.G., McCoy, A., et al. (2011). Overview of the ccp4 suite and current developments. Acta Crystallogr D Biol Crystallogr *67*, 235-42.
